# Application of Machine Learning Techniques for Predicting Compressive, Splitting Tensile, and Flexural Strengths of Concrete with Metakaolin

**DOI:** 10.3390/ma15155435

**Published:** 2022-08-07

**Authors:** Hammad Ahmed Shah, Qiang Yuan, Usman Akmal, Sajjad Ahmad Shah, Abdelatif Salmi, Youssef Ahmed Awad, Liaqat Ali Shah, Yusra Iftikhar, Muhammad Haris Javed, Muhammad Imtiaz Khan

**Affiliations:** 1 Department of Civil, Environmental and Ocean Engineering, Stevens Institute of Technology, Hoboken, NJ 07030, USA; 2 School of Civil Engineering, Central South University, Changsha 410075, China; 3 National Engineering Research Center of High-Speed Railway Construction Technology, Changsha 410075, China; 4 Department of Civil Engineering, University of Engineering and Technology, Lahore 54890, Pakistan; 5 School of Geosciences and Info-Physics, Central South University, Changsha 410075, China; 6 Department of Civil Engineering, College of Engineering, Prince Sattam Bin Abdulaziz University, Al-Kharj 16273, Saudi Arabia; 7 Structural Engineering Department, Faculty of Engineering & Technology, Future University in Egypt, New Cairo 11835, Egypt; 8 Department of Civil Engineering, University of Engineering & Technology Peshawar, Bannu 28100, Pakistan; 9 Department of Civil Engineering, CECOS University of IT and Emerging Sciences, Peshawar 25000, Pakistan

**Keywords:** gene expression programming, artificial neural network, M5P, random forest, metakaolin, compressive strength

## Abstract

The mechanical properties of concrete are the important parameters in a design code. The amount of laboratory trial batches and experiments required to produce useful design data can be decreased by using robust prediction models for the mechanical properties of concrete, which can save time and money. Portland cement is frequently substituted with metakaolin (MK) because of its technical and environmental advantages. In this study, three mechanical properties of concrete with MK, i.e., compressive strength (${f^{\prime }}_{c}$), splitting tensile strength ($f_{\mathrm{st}}$), and flexural strength (FS) were modelled by using four machine learning (ML) techniques: gene expression programming (GEP), artificial neural network (ANN), M5P model tree algorithm, and random forest (RF). For this purpose, a comprehensive database containing detail of concrete mixture proportions and values of ${f^{\prime }}_{c}$, $f_{\mathrm{st}}$, and FS at different ages was gathered from peer-reviewed published documents. Various statistical metrics were used to compare the predictive and generalization capability of the ML techniques. The comparative study of ML techniques revealed that RF has better predictive and generalization capability as compared with GEP, ANN, and M5P model tree algorithm. Moreover, the sensitivity and parametric analysis (PA) was carried out. The PA showed that the most suitable proportions of MK as partial cement replacement were 10% for FS and 15% for both ${f^{\prime }}_{c}$ and $f_{\mathrm{st}}$.

## 1. Introduction

Concrete is the second most-consumed substance in the world after water. Portland cement clinker is the base of the majority of cementitious binders that are being used in concrete. The production of cement consumes a huge amount of energy and releases about 7% of CO_2_ into the atmosphere [1]. During cement production, the calcination process, in which CaO is formed by extracting CO_2_ from CaCO_3_, is responsible for about 50% of CO_2_ production while the remaining 50% CO_2_ is produced by energy use [2]. The demand for cement is increasing and it was predicted that the annual usage of Portland cement would hit 6000 million tons by the year 2060 [3]. One of the ways to reduce cement consumption is to use industrial by-products or greener materials that use less energy during manufacturing as compared with cement, for example, metakaolin (MK). It was found that up to 170 kg of CO_2_ emission can be reduced per ton of cement production by using MK as a partial replacement for cement [4]. It is highly reactive pozzolan and reacts with calcium hydroxide to produce C-S-H and alumina-containing phases [5]. The use of MK in concrete as a partial replacement of cement helps to reduce the pore size distribution and improve the different mechanical properties [6]. It was found that MK helps to decrease the total porosity of paste by up to 20% [7] and refine pore structure [8].

The ${f^{\prime }}_{c}$ of concrete increased with the increase in MK content [9,10]. Kadri et al. [11] concluded that MK contributes to the mortar strength due to three factors: pozzolanic reaction of MK with calcium hydroxide, promotion of ordinary Portland cement hydration, and filler effect. The increase in ${f^{\prime }}_{c}$ with MK inclusion is also attributed to the fact that MK increases C-S-H gel and makes the structure dense [6]. Duan et al. [12] observed that the fine particles of MK fill gaps between hydration products, matrix and aggregates, and cement particles. This results in the denser interfacial transition zone (ITZ) between matrix and aggregates and pore structure and an increase in ${f^{\prime }}_{c}$. Moreover, the inclusion of MK reduces the needle-like ettringite crystals and increases the content of fibrous C-S-H and calcium aluminosilicate hydrates, which help to make the matrix denser. Ahmed et al. [13] found that the addition of 10–15% MK in concrete enhances the ${f^{\prime }}_{c}$ of concrete at both 28 and 60 days as compared to the control mix. Brooks et al. [9] observed that by increasing the content of MK from 0 to 10%, ${f^{\prime }}_{c}$ increased. Further increasing MK content to 15% lowers the ${f^{\prime }}_{c}$ but it was still higher than that of the control mix. As an incorporation of MK in cement-based materials increases ${f^{\prime }}_{c}$, it increases $f_{\mathrm{st}}$ as well, because Oluokun et al. [14] observed that an increase in ${f^{\prime }}_{c}$ generally reflected an increase in $f_{\mathrm{st}}$.

Madanoust et al. [15] investigated the effects of MK (0–20%) as cement replacement and with a water-to-binder (w/b) ratio of 0.32, 0.38, and 0.45 on the $f_{\mathrm{st}}$ of concrete. For w/b ratio of 0.32 and 0.45, $f_{\mathrm{st}}$ first increased by increasing MK and showed maximum value at 10% MK and then started to decrease but remained higher than the control mix. For concrete with a w/b ratio of 0.38, $f_{\mathrm{st}}$ increased until 15% MK and then started to decrease. Guneyisi et al. [16] replaced cement with 0–20% MK and investigated $f_{\mathrm{st}}$ of concrete at 1, 3, 7, 28, 90, and 120 days and with w/b ratios of 0.35 and 0.55. They observed that $f_{\mathrm{st}}$ of concrete increased by increasing MK content at all ages and for both w/b ratios. Dinakar et al. [17] tested $f_{\mathrm{st}}$ of concrete at specimen age of 28 days and w/b ratio of 0.3 and with MK (0–15%) as cement replacement. They observed that the optimum level of MK in terms of $f_{\mathrm{st}}$ was 10%. Lenka et al. [4] measured $f_{\mathrm{st}}$ of concrete with 0–20% MK as cement replacement at 7, 28, and 90 days and with a w/b ratio of 0.43. Similar to Dinakar et al. [17], they found 10% MK as the optimum level. As discussed earlier, an increase in ${f^{\prime }}_{c}$ generally reflects an increase in $f_{\mathrm{st}}$.

Lenka et al. [4] investigated the effect of 0 to 20% MK as cement replacement and observed FS at 7, 28, and 90 days. They pointed out that FS of concrete with MK at all replacement levels and at all ages was higher as compared with the control mix, and optimal content was found to be 10%. John et al. [18] observed an increase in FS of concrete by increasing MK replacement content and observed the maximum value of FS at 15% MK replacement. Vu et al. [19] investigated the incorporation of 10% MK in OPC and concluded that this percentage of cement replacement with MK performed satisfactorily in a normal and adverse environment. Tawfiq et al. [20] found satisfactory FS at 10% MK content.

Generally, MK incorporation improves the properties of mortar and concrete; therefore, the prediction of mechanical properties of concrete including ${f^{\prime }}_{c}$, $f_{\mathrm{st}}$, and FS could help to save time and cost, help in scheduling activities such as formwork removal, and promote the use of MK in the concrete industry. Researchers have been modelling different properties of cement-based materials for decades by using different machine learning (ML) techniques, including gene expression programming (GEP) [21], artificial neural network (ANN) [22], M5P model tree algorithm [23], and random forest (RF) [24].

GEP has been successfully used for predicting different concrete properties. For example, Javed et al. [21] developed a model for predicting ${f^{\prime }}_{c}$ of sugarcane bagasse ash concrete by using GEP and compared with linear regression (LR) and nonlinear regression (NLR) analysis. They concluded that the GEP technique performed better as compared with LR and NLR with a coefficient of determination (R^2^) of 0.83 and 0.85 for the training and testing sets, respectively. Aslam et al. [25] collected 357 data points from the literature and predicted the ${f^{\prime }}_{c}$ of high-performance concrete by using GEP. They compared the GEP model with LR, NLR, and other published models and concluded that GEP showed high performance as compared with other models with R^2^ values of 0.9 and 0.91 for training and testing sets, respectively. Azimi et al. [22] used the GEP and artificial neural network (ANN) to predict the ${f^{\prime }}_{c}$ and FS of cement mortar with micro- and nano-silica. They pointed out that ANN performed better as compared with GEP.

Naderpour et al. [26] predicted the ${f^{\prime }}_{c}$ of recycled aggregate concrete (RAC) by using ANN. The input parameters used were w/c, natural fine and coarse aggregates, water absorption, recycled coarse aggregate, and water/total material. ANN model gave R^2^ values of 0.9 for training, 0.89 for validation, and 0.83 for the testing set. Getahun et al. [27] estimated the ${f^{\prime }}_{c}$ and $f_{\mathrm{st}}$ of concrete incorporating rice husk ash as cement replacement and reclaimed asphalt pavement as natural aggregates replacement by using ANN. They observed a correlation coefficient (R) of 0.98. 0.99, and 0.98 for training, validation, and testing sets, respectively. Marijana et al. [28] used waste rubber as a natural aggregate replacement and predicted ${f^{\prime }}_{c}$ of concrete by ANN. They achieved an accuracy of 96% by changing ANN architecture. Mohammed et al. [29] modelled the ${f^{\prime }}_{c}$ of cement-based mortar with high volume fly ash by using ANN and M5P. For the training set, M5P gave 89% accuracy while ANN showed 93% accuracy. For the testing set, ANN and M5P showed values of R = 0.99 and 0.95, respectively.

Ayaz et al. [30] predicted the ${f^{\prime }}_{c}$ of concrete with a high volume of mineral admixtures such as fly ash and slag at different ages by using M5P. The main advantages of M5P are that it gives a simple empirical equation and it is convenient to develop and implement it [23]. Farooq et al. [31] completed a comparative study of the prediction of ${f^{\prime }}_{c}$ of high strength concrete (HSC) by using GEP and RF. RF (R^2^ = 0.96) performed better in prediction as compared with GEP (R^2^ = 0.90).

Previous studies have focused on the experimental route to find the optimum content of MK required to ensure desirable mechanical properties of concrete [15,16,17]. In the analysis of concrete structure, the key mechanical properties of concrete include ${f^{\prime }}_{c}$, $f_{\mathrm{st}}$, and FS. Therefore, it is desirable to develop a model which can accurately predict the ${f^{\prime }}_{c}$, $f_{\mathrm{st}}$, and FS of concrete with the following input parameters: cement (C), MK, w/b ratio, fine aggregate (FA), coarse aggregate (CA), superplasticizer (SP), and age of specimen (days). The following paragraph briefly summarizes the influence of input parameters on the mechanical properties of concrete.

Cement, which is the major binder of concrete, contains C_3_S and C_2_S that contribute to the formation of C-S-H. C-S-H makes up more than half of the cement paste and is the main factor in the development of concrete strength. For various kinds of concrete, the w/b ratio has varied effects. For example, in low- and medium-strength concrete, the porosity of ITZ and matrix increases by increasing the w/b ratio, thus deteriorating the mechanical properties. However, in high-strength concrete, a small reduction in the w/b ratio results in a high increase in the ${f^{\prime }}_{c}$. ITZ that is less porous and the hydration product’s large surface area at a low w/b ratio are responsible for this. As compared to other input parameters of concrete, aggregates contribute less to strength. The strength of the aggregate is scarcely used in normal strength concrete. The failure or capacity of concrete is typically caused by ITZ and matrix since the aggregate particles are several times stronger than these two components. The SP ensures the workability/rheology of concrete at a low w/b ratio, so by decreasing the w/b ratio, the strength increases. The age of a sample is another significant input component. Age improves hydration products. By increasing time, the hydration of anhydrous cement particles occurs, which increases the amount of C-S-H gel and increases strength [32].

This paper has two aims: (1) to model and compare the ${f^{\prime }}_{c}$, $f_{\mathrm{st}}$, and FS of concrete incorporating MK by an evolutionary algorithm (GEP), ANN, and decision trees (M5P and RF). (2) To explore the influence of different input parameters on the mechanical properties of concrete with MK by using parametric analysis. Moreover, sensitivity analysis was also carried out in order to find out the relative contribution of input parameters on mechanical properties. It is important to mention that the compressive strength of concrete with MK was not modelled by using M5P because of the large database (around 982 data points) which generates a significant number of linear models (greater than 40). More suitable ML techniques for the prediction of mechanical properties of concrete with MK may be found.

## 2. Data Collection

The database was collected from the literature and outliers were deleted. The remaining data comprised 982 data points for ${f^{\prime }}_{c}$, 204 data points for $f_{\mathrm{st}}$, and 63 data points for FS. Our aim was to collect a large database and, therefore, all test results of mechanical properties of concrete with MK conducted based on international standards were collected for use in this study. This database was collected from 45 published studies [4,15,16,17,18,33,34,35,36,37,38,39,40,41,42,43,44,45,46,47,48,49,50,51,52,53,54,55,56,57,58,59,60,61,62,63,64,65,66,67,68,69,70,71,72,73] and is shown in the supplementary document. In the supplementary document, Tables S1–S3 contain the information of input parameters and output for ${f^{\prime }}_{c}$, $f_{\mathrm{st}}$, and FS database, respectively. The collected database includes C, MK, w/b ratio, FA, CA, SP, and days as input parameters and ${f^{\prime }}_{c}$, $f_{\mathrm{st}}$, and FS as output parameters. The descriptive statistics of input and output variables used in the training set are shown in Table 1. These values help to give insights into the range and distribution-related properties of independent and dependent variables used in the training set.

All the data points used for modelling of ${f^{\prime }}_{c}$ of concrete with MK were for cube 150 mm. The ${f^{\prime }}_{c}$ of cylinder ∅100 mm×200 mm was converted to ${f^{\prime }}_{c}$ of cube 150 mm by multiplying with factor 1.1 for normal-strength concrete (NSC) and 0.98 for HSC (according to ACI 363R, HSC is concrete that has specific ${f^{\prime }}_{c}$ for the design of 55 MPa or greater). In addition, ${f^{\prime }}_{c}$ of cube 100 mm was converted to cube 150 mm by multiplying with 0.96 for NSC and 0.9 for HSC [74]. All the results of $f_{\mathrm{st}}$ were for cylinder ∅150 mm×300 mm. The $f_{\mathrm{st}}$ results obtained from cylinders ∅150 mm×150 mm and ∅100 mm×200 mm were converted into the equivalent of $f_{\mathrm{st}}$ obtained from cylinder ∅150 mm×300 mm by multiplying with factors 0.93 and 0.91, respectively [75,76]. The database for FS was for the prism size 100×100×500 mm.

## 3. Methodology

### 3.1. Gene Expression Programming

GEP is a branch of genetic programming (GP) and it was originated by Ferreira [77]. The GP is a method for solving problems that are not domain specific. It uses Darwinian reproduction and the survival of the fittest principles to solve problems. In order to obtain a solution whose length can change throughout a run, GP employs a parse tree structure. Function set, terminal set, fitness function, control parameters, and terminal condition are the five distinct elements of GEP. The first three components control the algorithm’s search space, while the latter two components control the search’s speed and quality [78]. A character string of fixed length is used to get a solution in the GEP algorithm. The parse trees of different sizes and shapes are then used to present the solution and these trees are called expression trees (ETs). The complex and nonlinear programs can be generated with the help of the multi-genic nature of GEP. Arithmetic operations are represented as a function set in each gene of the GEP (for example, +, −, ×, /, etc.), and constants and fixed-length variables are represented as a terminal set (for example, 1, 2, a, b). In general, the number of chromosomes controls how long the program will run. An error may be reduced but running time is lengthened by increasing the number of chromosomes.

**Figure 1:**
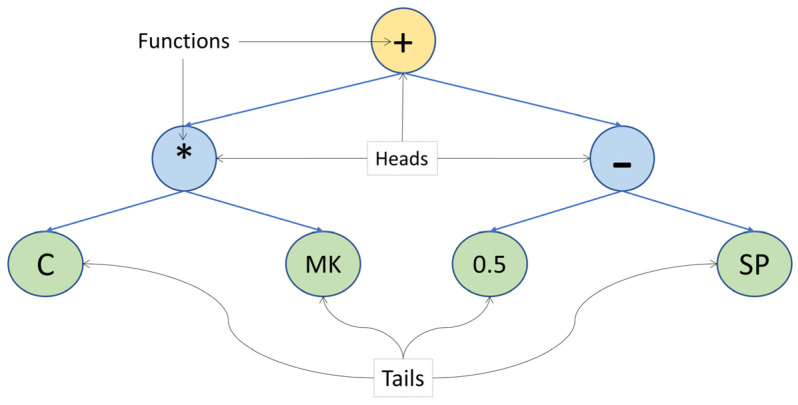
ET with one gene and three head size.

Figure 1 represents the ET with one gene, three head sizes, and a function set of +, −, and ∗. In order to obtain the mathematical formula, ET has to be read from left to right and top to bottom. The mathematical formula of ET in Figure 1 is ((C ∗ MK) + (0.5 − SP)).

**Figure 2:**
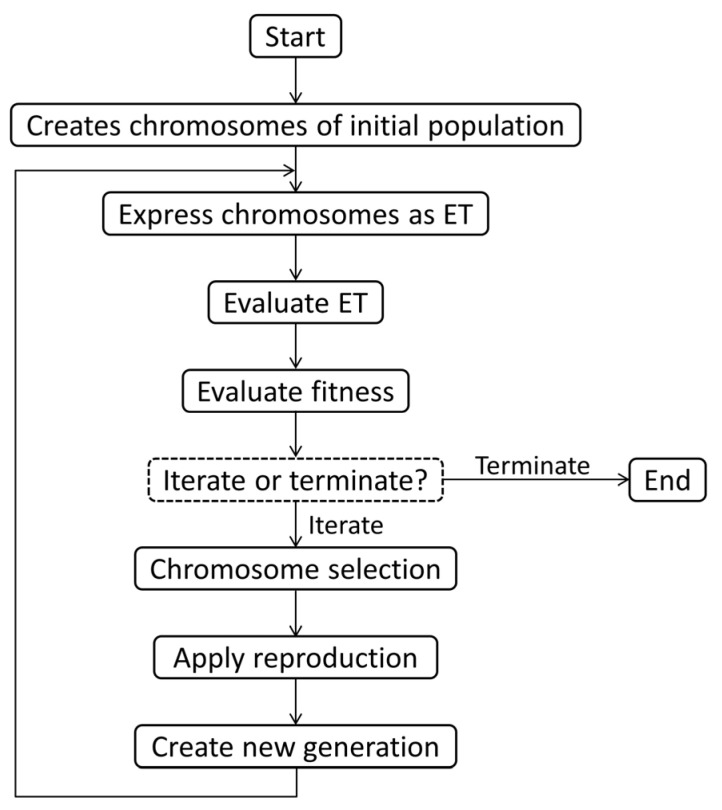
Flowchart of GEP algorithm (adapted with permission from [79]).

Figure 2 shows the flowchart of the GEP algorithm. Random generation of a chromosome with a fixed length for each individual is the starting point of the GEP algorithm. The individuals are evaluated and chosen based on their fitness for reproduction. This process continues with a new individual for a few generations and stops when a solution is found. Conversion in population is performed on the selected program by using genetic operators, such as mutation, rotation, and crossover [77].

### 3.2. Artificial Neural Network

Artificial neural network (ANN) is a widely used artificial intelligence (AI) method. There are many types of ANN such as radial basis function network, feedforward neural network (FNN), spiking neural network, etc. Among them, the widely used method is FNN [80]. Single-Layer Perceptron (SLP) and Multi-Layer Perceptron (MLP) are two types of FNN. Because of a single perceptron, SLP cannot execute non-linear problems; therefore, MLP is often used for non-linear problems [80].

**Figure 3:**
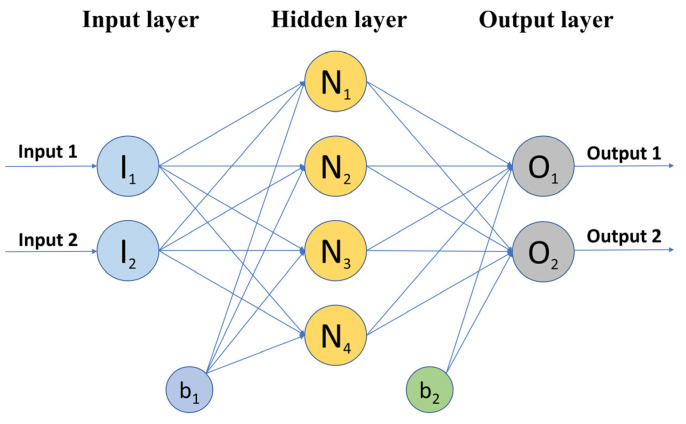
Schematic of three-layer MLP.

An input layer, hidden layer(s), units (neurons), weights, an activation function, and an output layer are the typical components of an MLP. The input layer receives information from the outside environment. Without completing any calculations, the input layer sends these data to neurons in the hidden layer. The majority of a network’s internal processing takes place in hidden layers, which are situated in between the input and output layers. The calculations are displayed to the environment outside by the output layer. The weights are used to connect adjoining layers and the function of the activation function is to decide how neurons will generate output value for the next layer [80]. Figure 3 shows a three-layer MLP with two inputs, one hidden layer with four hidden neurons, and two outputs.

Three common forms of activation functions are linear transfer (purelin), hyperbolic tangent (tanh), and sigmoid (logsig). For problems with function fitting, linear transfer functions work effectively. The ranges of the outputs of the tanh and logsig functions, respectively, are −1 to 1 and 0 and 1. The fact that tanh can simulate input values that contain negative, neutral, and positive numbers is one of its advantages over logsig [81].

There are a few advantages of ANN, such as: (1) it can model the relatively complex process and it does take outliers into account which makes its scope broad; (2) it can learn from examples and can build a relationship between dependent and independent variables [82]. More information about the ANN can be found in [81].

### 3.3. M5P Model Tree Algorithm

M5 algorithm was originally discovered by Quinlan [83] and the M5P algorithm [84] is its expanded form. In order to handle enumerated attributes and missing values for attributes, the M5P method was modified from M5. The M5P algorithm converts all enumerated properties into binary variables prior to tree creation [85].

**Figure 4:**
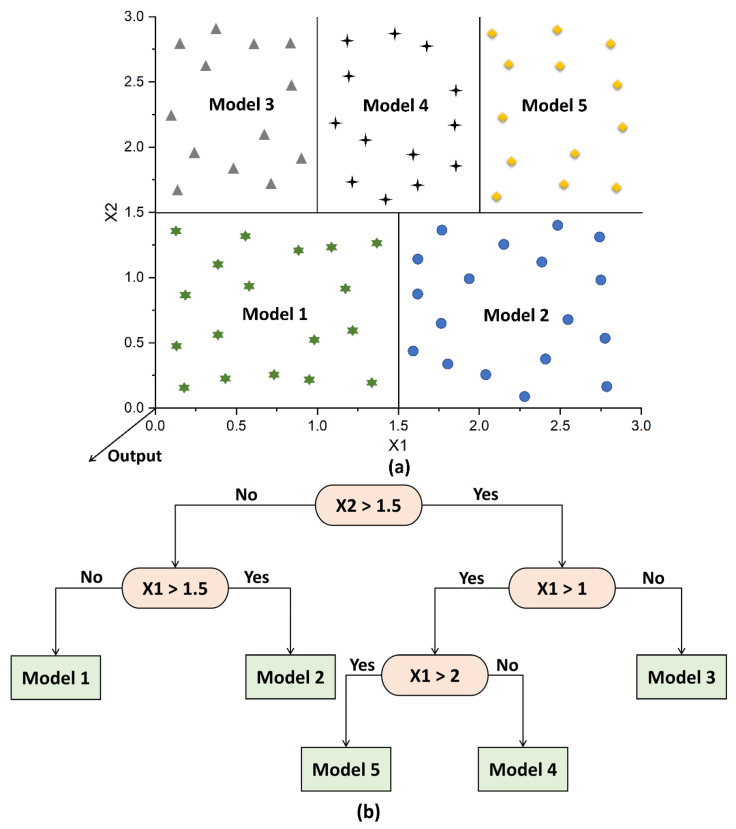
Illustration of M5 algorithm: (**a**) splitting of input space; (**b**) building of tree.

The illustration of the M5 algorithm is presented in Figure 4. The input data are divided into a number of sub-spaces, each of which contains data with shared characteristics (Figure 4a). To lessen a variation in the data inside a specific sub-space, linear regression models are applied. The splitting process is then carried out on a number of nodes depending on data gathered from the preceding stage, and each node is separated according to a certain attribute (Figure 4b). This stage enables the creation of a structure that resembles an inverted tree. When it comes to fresh data, they begin at the top of the tree’s root, travel through the nodes, and finally arrive at the leaf. Each node’s mathematical logic compares the data to the split value and aids in determining the data’s path to the leaf.

The input space is initially partitioned into a tree-like structure of different sub-spaces. At the node, the variability is calculated using the standard deviation of the values. Standard deviation reduction (*SDR*) is used to reduce the expected error at the node and helps to build a tree as follows:

(1) $$SDR=sd\left(S\right)-\underset{i}{\sum }\frac{S_{i}}{\left|S\right|}\times sd\left(S_{i}\right)$$

where sd is the standard deviation, $S_{i}$ are the sets produced by splitting node in accordance with a given attribute, and S is the set of data that comes to the node [84].

Using a pruning strategy, the over-training issue is managed. However, the trimming procedure might result in jarring breaks between adjacent linear models. The final phase is the smoothing procedure to solve this issue. The final model of the leaf is created during the smoothing phase by combining all models from the leaf to the root. This filters the estimated value of the leaf [23].

### 3.4. Random Forest

RF is a supervised ML method that comprises an ensemble of tree structures. ML techniques like bagging and random feature selection are used in RF [86]. In bagging, the bootstrap sample is generated using training data, and each tree is individually formed based on this sample. The estimate process then makes use of the average of the tree outputs [87]. A modified variation of bagging is RF. In RF, instead of selecting all features for a tree, a random subset of features is chosen. Due to its randomness, RF is resistant to overfitting and outperforms other ML methods like ANN and support vector machines [86].

**Figure 5:**
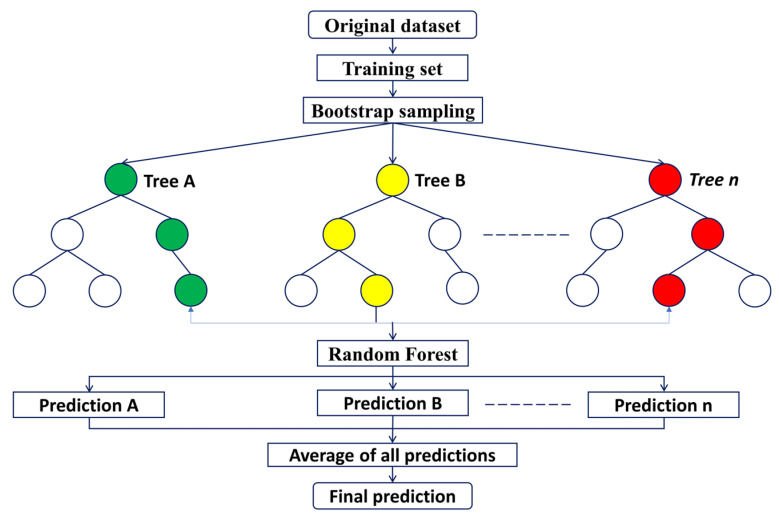
Illustration of random forest prediction.

RF has good generalization capability [86] and it provides a flexible framework with room for selecting objective functions (task-specific), various classes of splitting functions, or posterior models. Tree depth and the number of trees are the main hyperparameters in RF. The depth of a tree directly impacts the generalization ability of each tree, so its maximal allowable limit should be optimized [88]. An increasing number of trees helps to decrease prediction error by average out of noisy predictions. The schematic of the RF is shown in Figure 5. A detailed description of RF methodology can be found in [86].

## 4. Model Development and Evaluation Criteria

The collected database was randomly divided into two sets: 70% and 30% for the training and testing sets, respectively. The ${f^{\prime }}_{c}$, $f_{\mathrm{st}}$, and FS of concrete incorporating MK were considered to be a function of the following input parameters while developing models:

(2) $${f^{\prime }}_{c},\;f_{\mathrm{st}},\;\mathrm{and}\;\mathrm{FS}\;=\;f\;(C,\;\mathrm{MK},\;w/c,\;\mathrm{FA},\;\mathrm{CA},\;\mathrm{SP},\;\mathrm{days})$$

where ${f^{\prime }}_{c}$, $f_{\mathrm{st}}$, and FS are in MPa, while C, MK, FA, CA, and SP are in kg/m^3^.

For GEP modelling, three models were developed for the mechanical properties of concrete incorporating MK named: GEP I for ${f^{\prime }}_{c}$, GEP II for $f_{\mathrm{st}}$, and GEP III for FS. The parameters used in the GEP algorithm for three GEP models are shown in Table 2. The Sqrt, Exp, Ln, Log, Inv, X2, X3, X4, X5, 3Rt, 4Rt, 5Rt denote square root, exponential, natural logarithm, inverse, X to the power of 2, X to the power of 3, X to the power of 4, X to the power of 5, cube root, quartic root, and quintic root, respectively.

The input, output, and hidden layer specifications are the initial stage in creating an ANN model(s). All of the ANN models used in this study have one hidden layer, one input layer, and one output layer. All of the proposed ANN models have a 7-n-1 architecture. Trainlm, which changes bias and weight values in line with Levenberg-Marquardt (LM) optimization, was utilized for the training function [81]. Additionally, for the performance function and adaptation learning function, respectively, learngdm and mean squared error were used. The log-sigmoid was chosen as a transfer function in all created ANN models.

Three models were developed for the mechanical properties of concrete with MK named: ANN I for ${f^{\prime }}_{c}$, ANN II for $f_{\mathrm{st}}$, and ANN III for FS. In this study, the number of epochs, max_fail, μ, and min_grad values were kept as 1000, 35, 0.001, and 1 × 10^−7^, respectively for all developed ANN models.

The M5P algorithm generates linear regression mathematical equations after making different classes of data. The general form of the M5P algorithm can be written as follow:

(3) $${f^{\prime }}_{c},\;f_{\mathrm{st}},\;\mathrm{or}\;\mathrm{FS}\;=a+\left(b\times C\right)+\left(c\times \mathrm{MK}\right)+\left(d\times w/b\right)+\left(e\times \mathrm{FA}\right)+\left(f\times \mathrm{CA}\right)+\left(g\times \mathrm{SP}\right)+\left(h\times \mathrm{days}\right)$$

Two M5P-based models were developed for concrete with MK inclusion named M5P II for $f_{\mathrm{st}}$ and M5P III for FS. Similar to other GEP and ANN techniques used in this study, three models were developed for estimating the mechanical properties of concrete with MK by using RF named RF I for ${f^{\prime }}_{c}$, RF II for $f_{\mathrm{st}}$, and RF III for FS.

For comparison purposes among models developed by GEP, ANN, M5P, and RF, graphical presentations of absolute error (AE) were drawn for both training and testing data sets. A horizontal line on an absolute error of 10 MPa was drawn for ${f^{\prime }}_{c}$ results and the percentages of data below it were mentioned. For $f_{\mathrm{st}}$ and FS results, this operation was performed on an absolute error of 0.75 MPa.

For the four ML techniques, several trials were run in order to obtain a higher value of the R^2^ and R, and lower values of relative squared error (RSE), mean absolute error (MAE), root mean squared error (RMSE) for both training and testing sets. Moreover, a performance index (ρ) was used to assess the model performance as a function of both R and relative root mean squared error (RRMSE).

The mathematical expressions for R^2^, R, RSME, RRSME, MAE, RSE, and ρ are given in Equations (4)–(10).

(4) $$R^{2}=1-\left(\frac{\sum_{i=1}^{n}{\left(e_{i}-p_{i}\right)}^{2}}{\sum_{i=1}^{n}{\left(p_{i}\right)}^{2}}\right)$$

(5) $$R=\frac{\sum_{i=1}^{k}\left(e_{i}-{\;\bar{e}}_{i}\right)\left(p_{i}-{\;\bar{p}}_{i}\right)}{\sqrt{\sum_{i=1}^{k}{\left(e_{i}-{\;\bar{e}}_{i}\right)}^{2}\sum_{i=1}^{k}{\left(p_{i}-{\;\bar{p}}_{i}\right)}^{2}}}$$

(6) $$\mathrm{aRMSE}=\sqrt{\frac{1}{n}\sum_{i=1}^{n}{\left(e_{i}-p_{i}\right)}^{2}}$$

(7) $$\mathrm{RRMSE}=\frac{1}{\left|\;\bar{e}\right|}\sqrt{\frac{1}{n}\sum_{i=1}^{n}{\left(e_{i}-p_{i}\right)}^{2}}$$

(8) $$\mathrm{RSE}=\frac{\sum_{i=1}^{n}{\left(p_{i}-e_{i}\right)}^{2}}{\sum_{i=1}^{n}{\left(\;\bar{e}-e_{i}\right)}^{2}}$$

(9) $$\mathrm{MAE}=\frac{\sum_{i=1}^{n}\left|e_{i}-p_{i}\right|}{n}$$

(10) $$\rho =\frac{\mathrm{RRMSE}}{1+R}$$

where $\bar{e}$ is the average experimental value, n represents the total number of samples, and $e_{i}$ and $p_{i}$ are the experimental and predicted values, respectively.

Poor performance is indicated for models with R^2^ < 0.7 [89] while a model with R > 0.8 indicates a significant positive correlation between estimated and experimental outcomes [90]. The RSME, MAE, and RSE illustrate how accurate the proposed model is; a high value demonstrates how far the estimated results differ from the experimental results, whilst a low value demonstrates an acceptable level of accuracy in the estimated outcomes.

In addition to the aforementioned statistical indicators, the discrepancy ratio (DR) was also utilized to evaluate the performance of developed models. DR is expressed as:

(11) $$\mathrm{DR}=\log\frac{p_{i}}{e_{i}}$$

where all the terms are as described previously.

A precise match between the estimated and real values is indicated by a DR of zero. Between actual and predicted values, a negative DR denotes underestimation and a positive DR denotes overestimation. In this study, in models developed for ${f^{\prime }}_{c}$ of concrete with MK, the accuracy is defined as a percentage of DR values fall in the range of −0.1 to 0.1; this range was also used by Benhood et al. [23]. However, for $f_{\mathrm{st}}$ and FS database, the accuracy is defined as percentage of DR values that fall in the range of −0.05 to 0.05.

## 5. Results and Discussion

### 5.1. Developed Models for Compressive Strength

#### 5.1.1. GEP I Model

**Figure 6:**
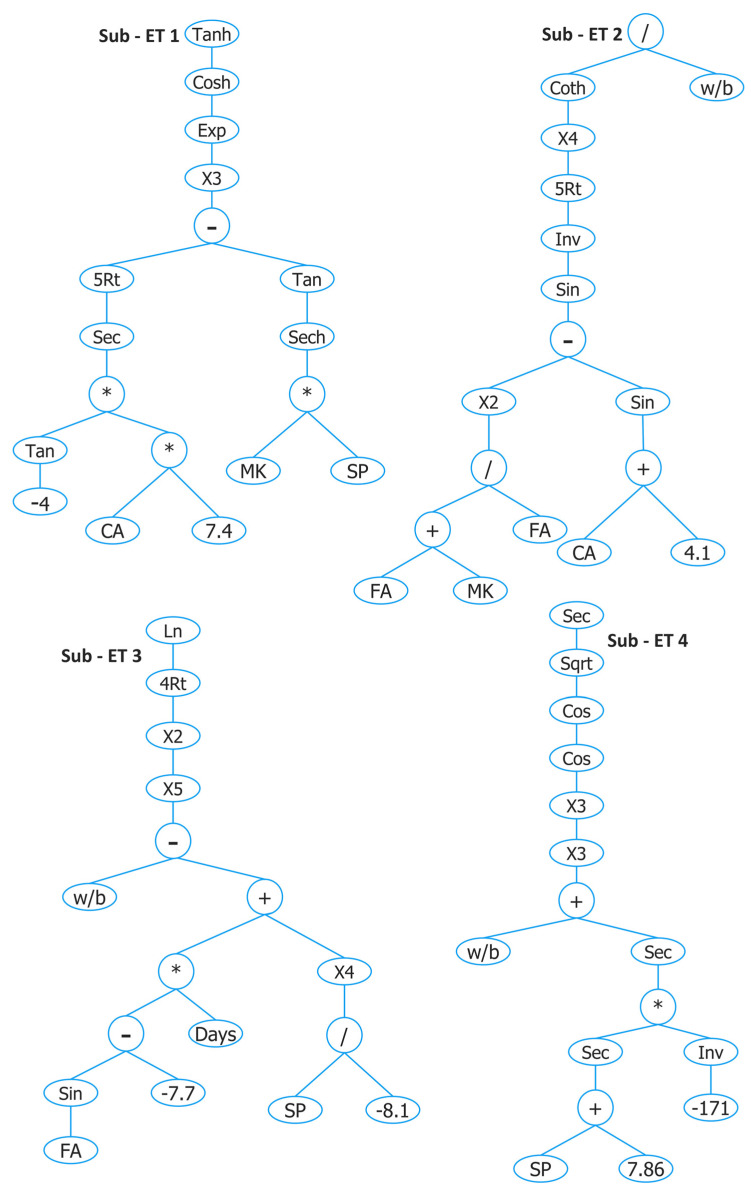
Expression tree of GEP I developed for predicting ${f^{\prime }}_{c}$ of concrete with MK.

To develop all GEP models in this study, GeneXproTools 5.0 software was used. Different researchers have used different parameters of the GEP algorithm in order to obtain a model with high accuracy and generalization capability as shown in Table 3. In this study, several runs were tried in order to obtain: (1) a relatively simple model by trying to minimize the number of genes and head size as a number of genes increases sub–ET size and head size increases the complexity within each gene. (2) A model that gives a high value of R^2^ and R for training and testing sets and a low value of MAE, RMSE, RSE, and ρ. After several trials, the parameters of the optimum GEP model (GEP I) for ${f^{\prime }}_{c}$ of concrete with MK are given in Table 2 and generated ETs are shown in Figure 6. The mathematical formula was obtained from Figure 6 by following the procedure as mentioned in Section 3.1 and given as follows:

(12) $${f^{\prime }}_{c}=A\;\times \;B\;\times \;C\;\times \;D$$

where

$$A=\mathrm{Tanh}\;\left(\cosh\left(\exp(\left(\sqrt[{5}]{\sec\left(-0.52\mathrm{CA}\right)}\right)-{\left(\tan\left(\mathrm{sech}\left(\mathrm{MK}\times \mathrm{SP}\right)\right)\right)}^{3}\right))\right)\;B=\frac{\mathrm{Coth}\;{\left(\sqrt[{5}]{\frac{1}{\sin\left(\left({\left(\frac{\mathrm{FA}+\mathrm{MK}}{\mathrm{FA}}\right)}^{2}\right)-\left(\sin\left(4.1+\mathrm{CA}\right)\right)\right)}}\;\right)}^{4}}{w/b}\;C=\mathrm{Ln}\;\left(\sqrt[{4}]{{\left({\left(\left(\frac{w}{b}\right)-\left(\left(\left(\sin\left(\mathrm{FA}\right)+7.7\right)\times \left(\mathrm{Days}\right)\right)+\left({\left(\frac{\mathrm{SP}}{-8.1}\right)}^{4}\right)\right)\right)}^{5}\right)}^{2}}\;\right)\;D=\mathrm{Sec}\;\sqrt[{2}]{\cos(\cos{\left({\left(\frac{w}{b}+\sec((\sec\left(\mathrm{SP}+7.86)\right)\times \left(-\frac{1}{171}\right)\right)}^{3})\right)}^{3})}$$

**Figure 7:**
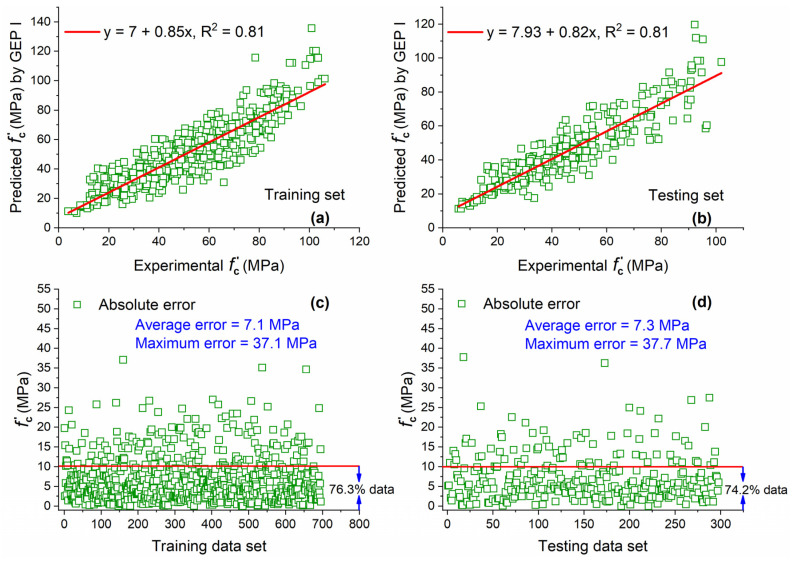
Experimental and predicted values of ${f^{\prime }}_{c}$ by GEP I for (**a**) training set and (**b**) testing set, and their corresponding absolute error for (**c**) training data set and (**d**) testing data set.

The comparison of actual and estimated results for training and testing data sets by GEP I for ${f^{\prime }}_{c}$ of concrete with MK and their absolute error is shown in Figure 7. The R^2^ value of 0.81 for the training set shows that GEP predicted values correlate well nonlinearly with actual results, while the R^2^ value of 0.81 for the testing set indicates that the GEP algorithm can predict the output well by using unseen data as input, indicating its high generalization capability.

AE was also plotted in order to assess the performance of GEP prediction. It is shown in Figure 7c that a relatively large portion of training data (76.3%) are below the AE of 10 MPa with an average error or MAE of 7.1 MPa. For testing data, 74.2% of data lie below 10 MPa with an average error of 7.3 MPa. Training data that are below 10 MPa AE are slightly higher (2.8%) as compared with testing data.

#### 5.1.2. ANN I Model

**Figure 8:**
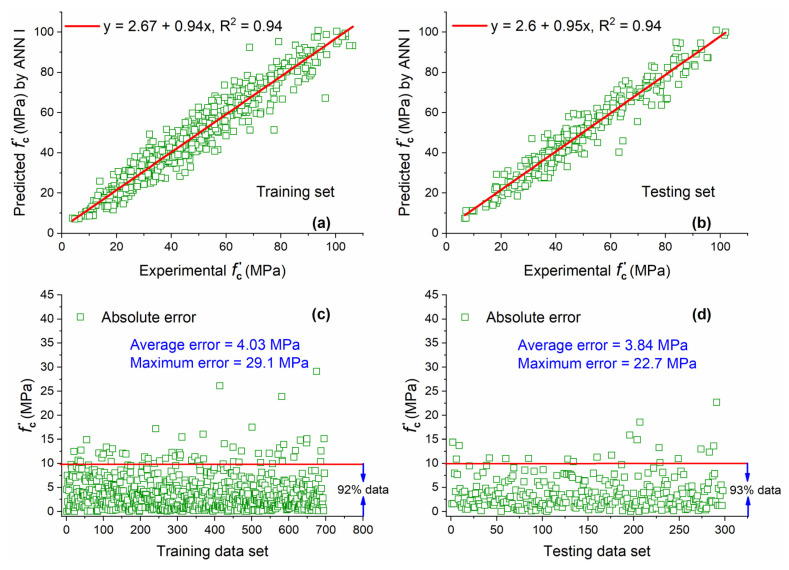
Experimental and predicted values of ${f^{\prime }}_{c}$ by ANN I for (**a**) training set and (**b**) testing set, and their corresponding absolute error for (**c**) training data set and (**d**) testing data set.

The ANN I model and other ANN models (ANN II and ANN III) developed in this study were trained by using MATLAB R2019a neural network toolbox. After several trials, the best ANN model was achieved with the architecture of 7-12-1 named ANN I. Figure 8a,b show that the value predicted by the ANN I model matches excellently with actual experimental results, having a value of R^2^ equal to 0.94 for both training and testing sets. This value of R^2^ for both data sets is 16% higher as compared with the value obtained from the GEP I model. The high R^2^ value for the testing set indicates that ANN has a high generalization capacity and can predict the output based on unseen data once it is adequately trained on given input parameters and output. Moreover, the slope of regression for both data sets is close to the ideal fit (1 for the ideal case) indicating a slight difference between actual and estimated results.

A very large portion of the training (92%) and testing (93%) data sets is below the AE of 10 MPa as shown in Figure 8c,d. The percentage of data below AE of 10 MPa obtained by ANN I is 20.6% and 25% higher for training and testing data sets as compared with GEP I. This large quantity of data below AE of 10 MPa shows that difference between actual and predicted results is small.

#### 5.1.3. RF I

**Figure 9:**
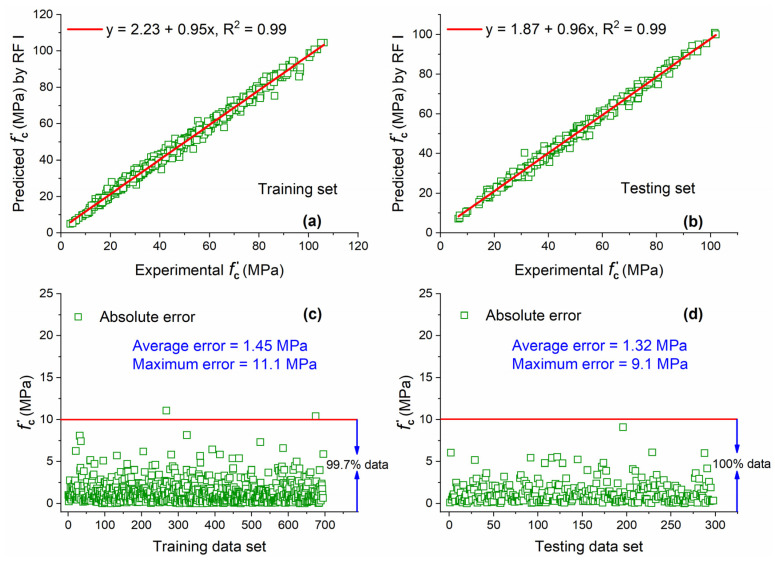
Experimental and predicted values of ${f^{\prime }}_{c}$ by RF I for (**a**) training set and (**b**) testing set, and their corresponding absolute error for (**c**) training data set and (**d**) testing data set.

The RF I model and other RF models in this study (RF II and RF III) were developed by using WEKA version 3.9.5 (developed by University of Waikato, Hamilton, New Zealand). All the settings of parameters were kept as default for all the RF models developed in this study. Figure 9a,b present the comparison between predicted values obtained from RF I and experimental results for training and testing sets. An excellent value of R^2^ (i.e., 0.99), which is approaching ideal condition 1, for both data sets indicates that RF is an excellent tool for the prediction of ${f^{\prime }}_{c}$ of concrete with MK and has a high capability to forecast output based on un-seen data set. The high prediction capability of RF I is also obvious from the slope of the regression line which is 0.95 and 0.96 for training and testing data sets, respectively. For both training and testing sets, the R^2^ values given by RF I are 22.2% and 5.3% higher than that of GEP I and ANN I, respectively.

The percentages of data of AE below 10 MPa are almost 100% for both data sets as shown in Figure 9c,d. This depicts that the difference between actual and predicted results is not high and is less as compared with GEP I and ANN I. This is also clear from the value of average error for both data sets which is 1.45 MPa for training and 1.32 MPa for the testing set.

#### 5.1.4. Comparison of GEP I, ANN I, and RF I

It is clear from Table 4 that the RF I predicted values correlated excellently with actual results, with values of R equal to 0.997 and 0.996 for training and testing sets, respectively. With respect to the values of R for both data sets, the order of correlation between predicted and actual results for developed models was RF I > ANN I > GEP I. In case of statistical errors (i.e., RMSE, RRMSE, RSE) and ρ value, the order of developed models for ${f^{\prime }}_{c}$ was RF I < ANN I < GEP I for the training data set. This shows that the RF I model has high performance (as indicated by the low value of ρ) and predicted results are close to experimental data followed by ANN I and GEP I. In the case of the testing set, for values of RRMSE, RSE, and ρ, the order of developed models is similar to that observed in the training set.

**Figure 10:**
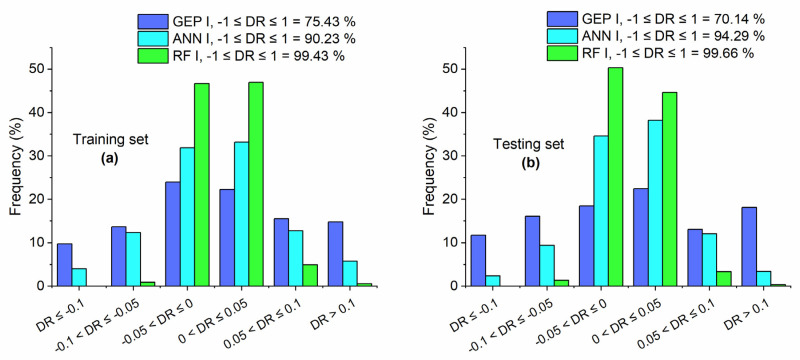
DR values of GEP I, ANN I, and RF I for (**a**) training set and (**b**) testing set.

In addition, Figure 10 shows that, for the training set, the accuracy of RF I, ANN I, and GEP I is 99.43%, 90.23%, and 75.43%, respectively, as measured by the percentage of DR values that fall in the range of −0.1 to 0.1. In the case of the testing set, the accuracy is 99.66%, 94.29%, and 70.14% for RF I, ANN I, and GEP I, respectively.

The high performance and prediction capability of ANN I over GEP I is in agreement with Nazari et al. [93] and Yu et al. [94]. Nazari et al. [93] predicted the water absorption (%) of HSC containing TiO_2_ nanoparticles by using two ANN-based models (they named ANN I and ANN II) and two GEP-based models (GEP I and GEP II). For the training set, ANN I and ANN II showed the values of R^2^ equal to 0.99 and 0.97, respectively, and GEP I and GEP II gave the values of R^2^ equal to 0.91 and 0.85, respectively. In the case of the testing set, ANN I and GEP I gave the value of R^2^ equal to 0.96 and 0.9, respectively, while these values were 0.93 and 0.85 in the case of ANN II and GEP II, respectively. Yu et al. [94] predicted the degradation of elastic modulus induced by the alkali-silica reaction by using soft computing techniques, including ANN and GEP. For the training set, ANN and GEP showed values of R equal to 0.98 and 0.86, respectively. For the testing set, these values were 0.93 and 0.91 by using ANN and GEP, respectively.

The high prediction and generalization capability of RF I as compared with GEP I is in agreement with the study by Mohsin et al. [95]. They modelled the ${f^{\prime }}_{c}$ of fly ash based geopolymer concrete by using GEP and RF. In case of RF model, the values of R for training and testing set were 0.98 and 0.99, respectively, while in case of the GEP model, these values were 0.86 and 0.96 for the training and testing sets, respectively.

### 5.2. Developed Models for Splitting Tensile Strength

#### 5.2.1. GEP II

**Figure 11:**
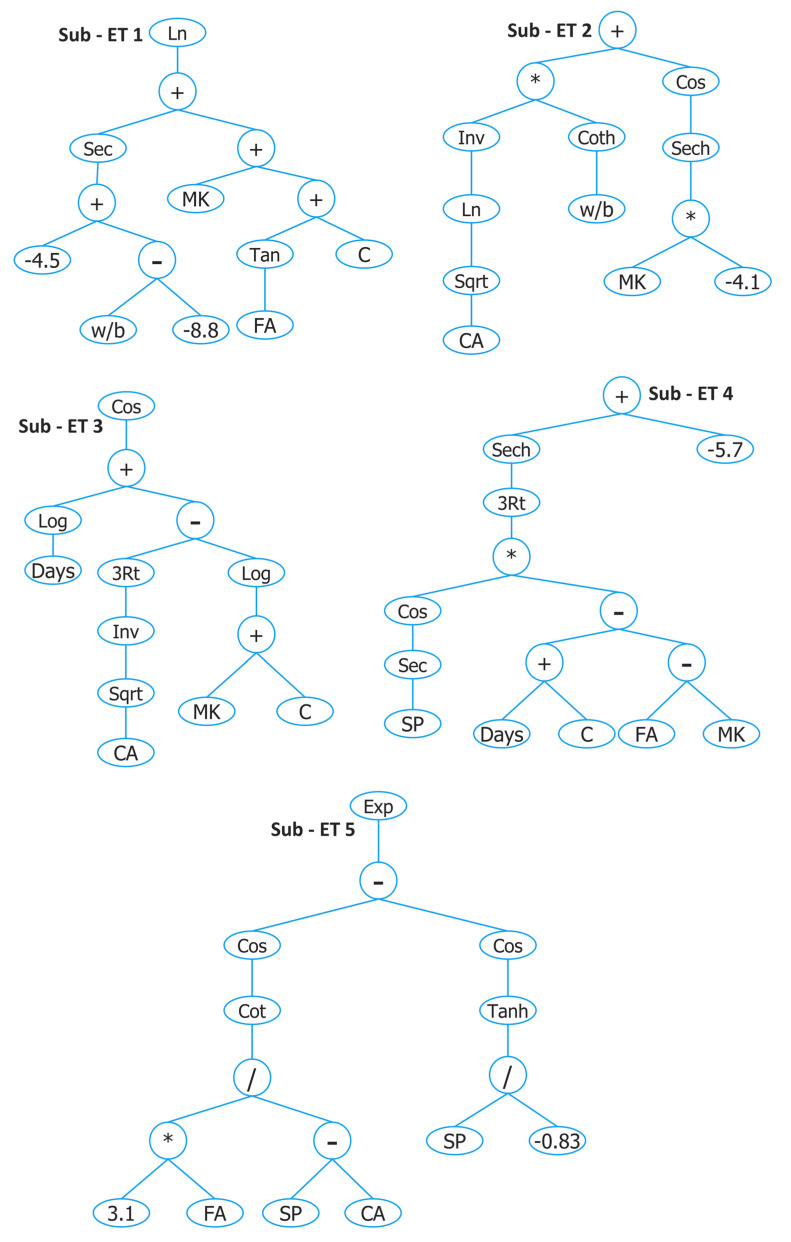
Expression tree of GEP II developed for predicting $f_{\mathrm{st}}$ of concrete with MK.

The parameters of the optimal GEP model (GEP II) are shown in Table 2 and the developed expression tree is given in Figure 11. The empirical expression decoded from the expression tree is given as follows:

(13) $$f_{\mathrm{st}}=A+B+C+D+E$$

where

$$A=\mathrm{Ln}\;(\sec\left(-4.5+\left(\frac{w}{b}+8.8\right)\right)+\left(\mathrm{MK}+\left(\tan\left(\mathrm{FA}\right)+C\right)\right))\;B=\left(\mathrm{Inv}\left(\ln\left(\sqrt[{2}]{\mathrm{CA}}\right)\right)\times \left(\coth\left(\frac{w}{b}\right)\right)\right)+\cos\left(\mathrm{sech}\left(-4.1\times \mathrm{MK}\right)\right)\;C=\mathrm{Cos}\;\left(\log\left(\mathrm{Days}\right)\right)+\left(\sqrt[{3}]{\mathrm{inv}\left(\sqrt[{2}]{\mathrm{CA}}\right)}-\log\left(\mathrm{MK}+C\right)\right)\;D=\mathrm{Sech}\left(\sqrt[{3}]{\cos\left(\sec\left(\mathrm{SP}\right)\right)\times (\left(\mathrm{Days}+C\right)-\left(\mathrm{FA}-\mathrm{MK})\right)}\right)-5.7\;E=Exp\;\left(\cos\left(\cot\frac{3.1\times \mathrm{FA}}{\mathrm{SP}-\mathrm{CA}}\right)-\cos\left(\tanh\left(\frac{\mathrm{SP}}{-0.83}\right)\right)\right)$$

**Figure 12:**
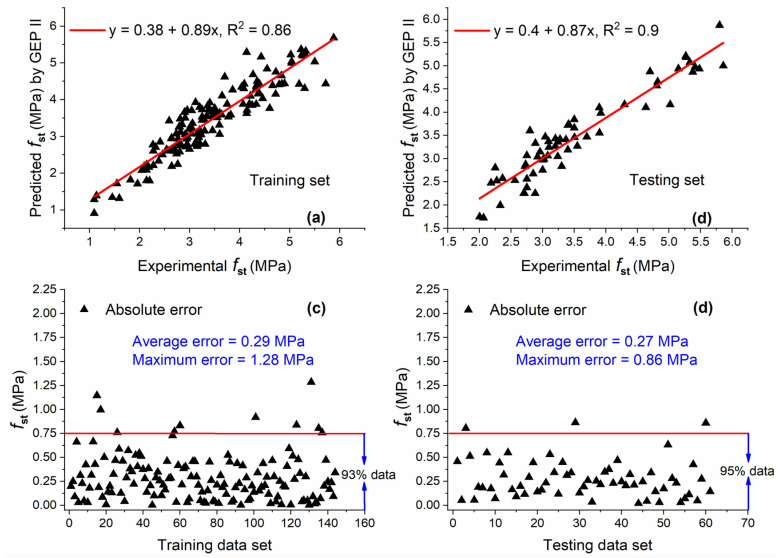
Experimental and predicted values of $f_{\mathrm{st}}$ by GEP II for (**a**) training set and (**b**) testing set, and their corresponding absolute error for (**c**) training data set and (**d**) testing data set.

The comparison of GEP II predicted values and actual results along with AE for both training and testing sets is shown in Figure 12. A high correlation between estimated and experimental values was found as indicated by R^2^ values which were 0.86 and 0.9 for training and testing sets. A slight difference of 0.04 was also observed between R^2^ values of training and testing sets. The slope of the regression line is high, i.e., 0.89 and 0.87 for training and testing sets, indicating a high correlation.

In addition, AE is plotted and the horizontal line is drawn on AE of 0.75 MPa, and the percentage of data below this line is shown in order to give more insight into the capability of the model to predict output close to real values as shown in Figure 12c,d. It is clear from the figures that the majority of data are below the AE of 0.75 MPa for both data sets (i.e., 93% and 95% for training and testing sets, respectively).

#### 5.2.2. ANN II

**Figure 13:**
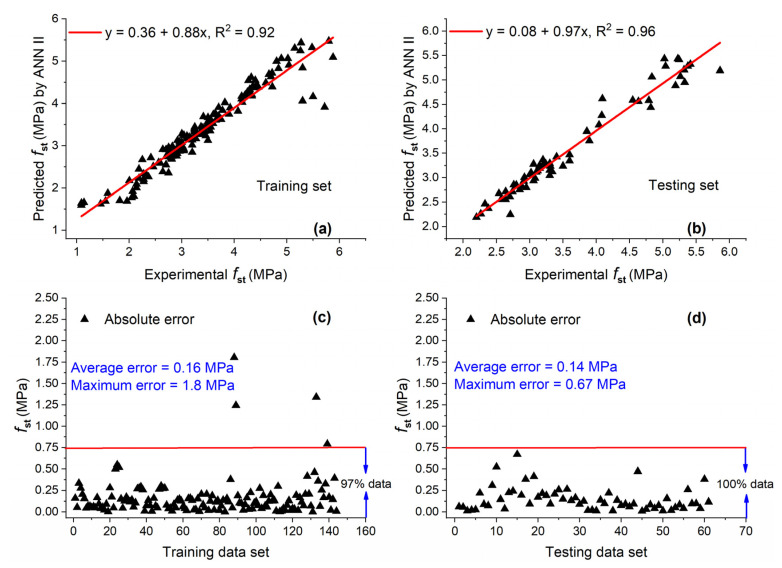
Experimental and predicted values of $f_{\mathrm{st}}$ by ANN II for (**a**) training set and (**b**) testing set, and their corresponding absolute error for (**c**) training data set and (**d**) testing data set.

After several trials, the best ANN model (ANN II) was obtained with an architecture of 7-11-1. The plot of predicted and actual values for the training and testing sets are shown in Figure 13a,b. Values of R^2^ are relatively close to 1 for training (R^2^ = 0.92) and testing (R^2^ = 0.96) sets. These higher values of R^2^ for both data sets indicate that ANN can recognize the relationship between input and output variables well and produce output by using unseen data with high accuracy. As compared with GEP II, these values were 6.98% and 6.7% higher for training and testing sets, respectively. Moreover, the slope of the regression line was slightly higher for the training set and considerably higher for the testing set as compared with GEP II.

Very large data are below the AE of 0.75 MPa for the training set (97%) and testing set (100%), as shown in Figure 13c,d. This shows that ANN II was trained well and predicted the output which was very close to the experimental output.

#### 5.2.3. M5P II

**Figure 14:**
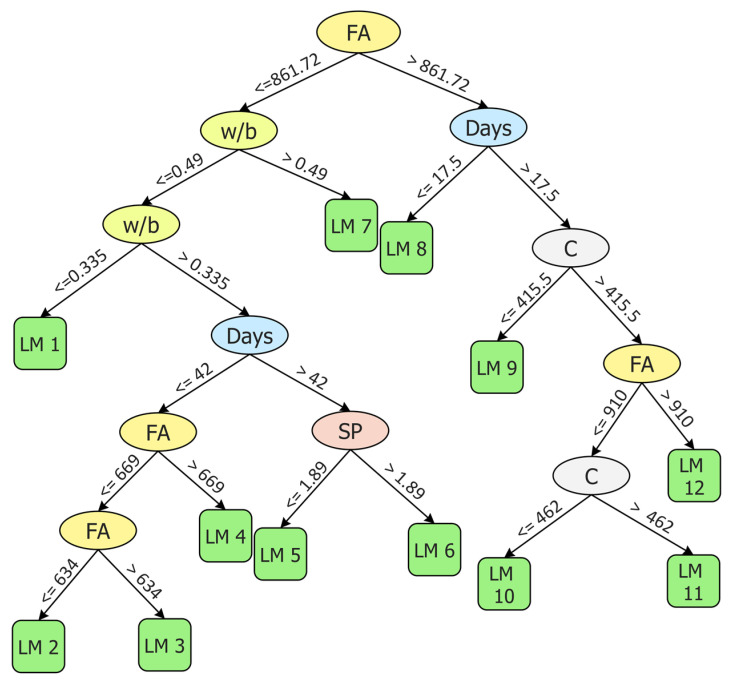
Generated model tree structure of M5P II.

In this study, all M5P models were developed by using the Waikato Environment for Knowledge Analysis (WEKA) software version 3.9.5 (Hamilton, New Zealand). The model trees are generated as shown in Figure 14. The term LM at the tree leaves represents the linear model identified by the M5P algorithm. The corresponding coefficients for linear models developed by M5P II based on Equation (3) are shown in Table 5.

**Figure 15:**
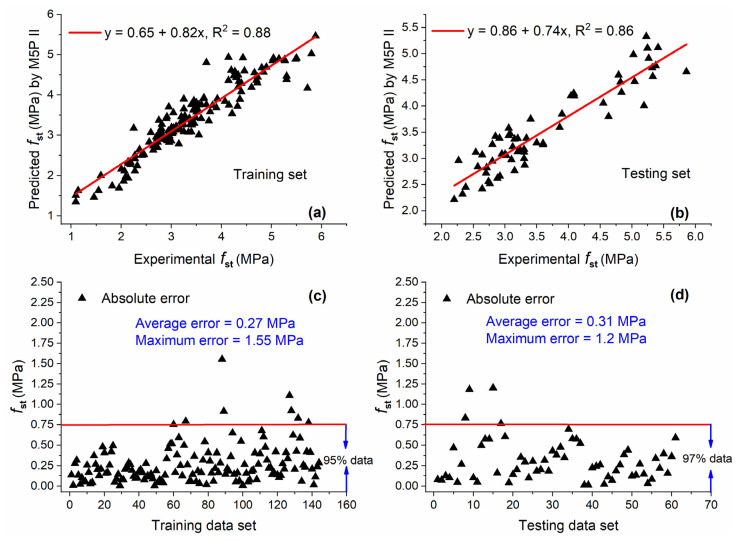
Experimental and predicted values of $f_{\mathrm{st}}$ by M5P II for (**a**) training set and (**b**) testing set, and their corresponding absolute error for (**c**) training data set and (**d**) testing data set.

Figure 15a,b depict the correlation between M5P II predicted values and experimental results. The values of R^2^ were 0.88 and 0.86 for training and testing sets, respectively. For the training set, this value was slightly higher as compared with GEP II but lower than that of ANN II. In the case of the testing set, it was lower than both GEP II and ANN II. In term of the percentage of data below AE of 0.75 MPa, M5P II perform slightly better as compared with GEP II for both training and testing sets.

#### 5.2.4. RF II

**Figure 16:**
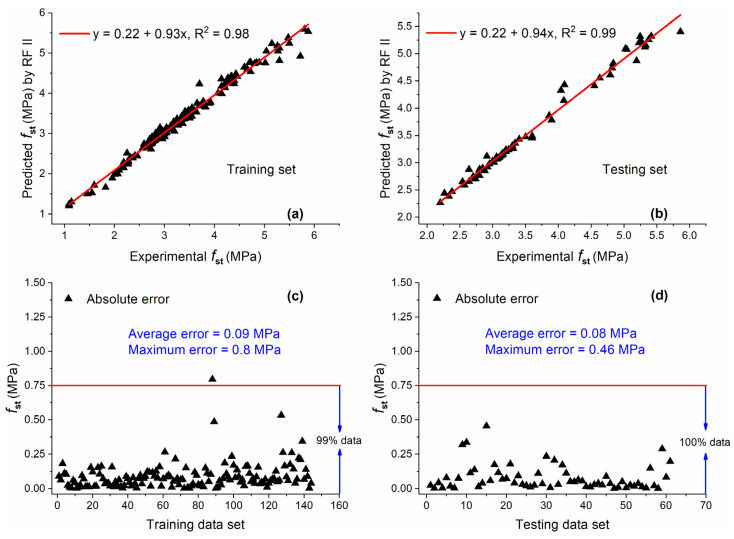
Experimental and predicted values of $f_{\mathrm{st}}$ by RF II for (**a**) training set and (**b**) testing set, and their corresponding absolute error for (**c**) training data set and (**d**) testing data set.

Figure 16 shows the comparison of actual and predicted values by RF II along with AE for training and testing sets. Excellent correlation was observed for both data sets, with R^2^ equal to 0.98 and 0.99 for training and testing sets. This impressive value of R^2^ for both data sets indicates that RF is a potential candidate for predicting $f_{\mathrm{st}}$ of concrete with MK with high accuracy and generalization capability. Moreover, almost all the data were below the AE of 0.75 MPa for both data sets. The average error for both data sets was approaching zero, indicating that the difference between actual and predicted values was very small.

For the training set, the values of R^2^ obtained by RF II were 13.95%, 6.5%, and 11.4% higher as compared with GEP II, ANN II, and M5P II, respectively. In the case of the testing set, R^2^ of RF II was 10%, 3.1%, and 15.11% higher than that of GEP II, ANN II, and M5P II, respectively.

#### 5.2.5. Comparison of GEP II, ANN II, M5P II, and RF II

**Figure 17:**
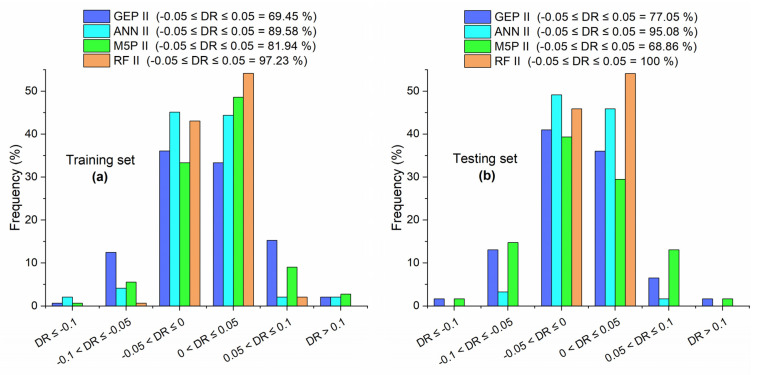
DR values of GEP II, ANN II, M5P II, and RF II for (**a**) training set and (**b**) testing set.

Table 6 shows statistical measures of different models developed for predicting $f_{\mathrm{st}}$ of concrete with MK. For the training set, the order of value of R for different models was RF II > ANN II > M5P II > GEP II. In the case of the value of RMSE, RRMSE, RSE, and ρ , this order was reversed, i.e., RF II < ANN II < M5P II < GEP II. A slight change was observed in the case of the testing set, in which M5P II and GEP II replaced each other’s position, respective to their positions in the case of the training set. In addition, Figure 17 shows that the accuracy of GEP II, ANN II, M5P II, and RF II is 69.45%, 89.58%, 81.94%, and 97.23%, respectively for the training set, while it is 77.05%, 95.08%, 68.86%, and 100%, respectively for the testing set. The order of accuracy given by DR for all models in Figure 17 is aligned with the order of accuracy of all models by using different statistical measures as shown in Table 6. Overall, RF performed better as compared with the other three machine learning techniques for predicting $f_{\mathrm{st}}$ of concrete with MK and these results are in agreement with the prediction results of ${f^{\prime }}_{c}$ database.

### 5.3. Developed Models for Flexural Strength

#### 5.3.1. GEP III

**Figure 18:**
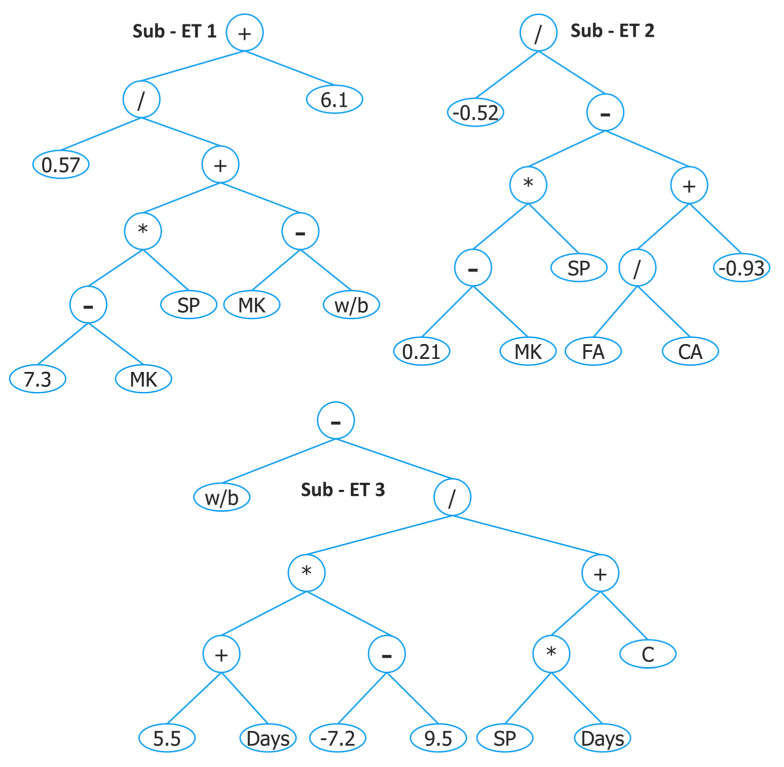
Expression tree developed by GEP III.

The optimal parameters found after several trials for predicting FS of concrete with MK are shown in Table 2 with the name GEP III. The ET developed by GEP III is given in Figure 18 and decoded mathematical equation from the figure is as follows:

FS = A + B + C(14)

where

$$A=\left(\frac{0.57}{\left(\left(7.3-\mathrm{MK}\right)\times \left(\mathrm{SP}\right)\right)+\left(\mathrm{MK}-w/b\right)}\right)+6.1\;B=\frac{-0.52}{\left(\left(0.21-\mathrm{MK}\right)\times \left(\mathrm{SP}\right)\right)-\left(\left(\frac{\mathrm{FA}}{\mathrm{CA}}\right)-0.93\right)}\;C=\frac{w}{b}-\frac{\left(5.5+\mathrm{Days}\right)\times \left(-16.7\right)}{\left(\mathrm{SP}\times \mathrm{Days}\right)+C}$$

**Figure 19:**
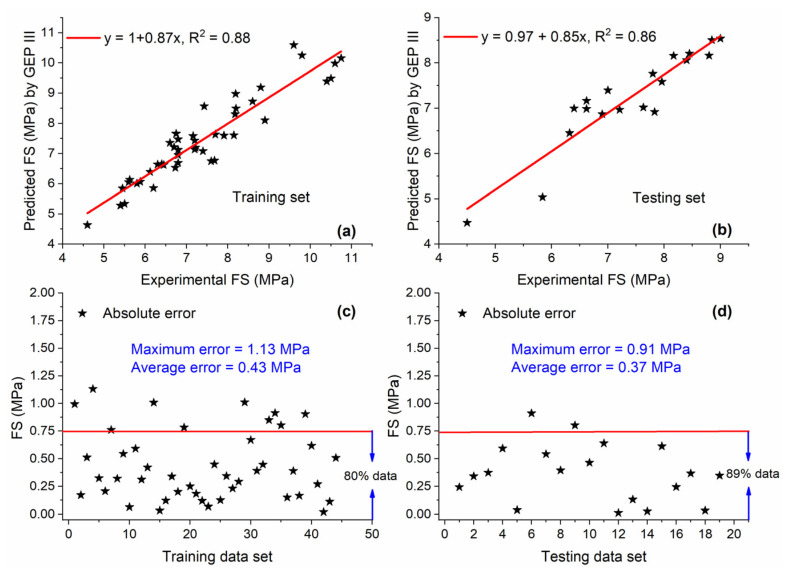
Experimental and predicted values of FS by GEP III for (**a**) training set and (**b**) testing set, and their corresponding absolute error for (**c**) training data set and (**d**) testing data set.

The relationship between GEP III predicted values and actual values along with AE for both training and testing sets is shown in Figure 19. The values of R^2^ were 0.88 and 0.86 for training and testing sets, respectively. In the case of the training set, 80% of data are below AE of 0.75 MPa, while it is 89% in the case of the testing set, as shown in Figure 19c,d.

#### 5.3.2. ANN III

**Figure 20:**
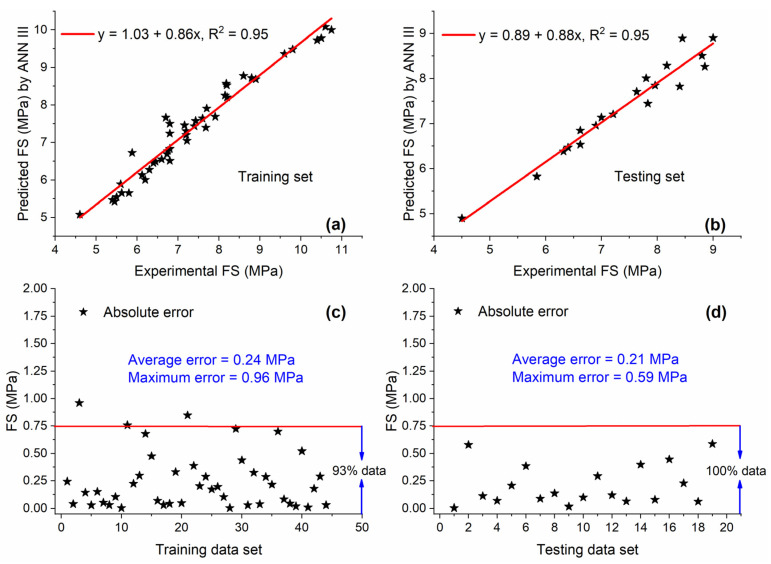
Experimental and predicted values of FS by ANN III for (**a**) training set and (**b**) testing set, and their corresponding absolute error for (**c**) training data set and (**d**) testing data set.

Similar to ANN II, the best accuracy for ANN III was obtained with the architecture of 7-11-1. As shown in Figure 20a,b, the value of R^2^ = 0.95 for both training and testing data sets shows that ANN III trained well with given inputs and outputs and estimated outputs by using unseen input parameters with high accuracy. This value of R^2^ was 7.95% and 10.5% higher as compared with training and testing sets of GEP III, respectively. Significantly less error was noted in the case of the testing set as 100% of data are below the AE of 0.75 MPa, showing the high generalization ability of ANN III, as shown in Figure 20d.

#### 5.3.3. M5P III

By using the default setting for parameters of WEKA software, only one linear model was obtained (due to the small database) for FS whose empirical expression is given below:

(15) $$\mathrm{FS}=16.33-\left(0.0145\times C\right)-\left(10.8\times \frac{w}{b}\right)+\left(0.33\times \mathrm{days}\right)$$

**Figure 21:**
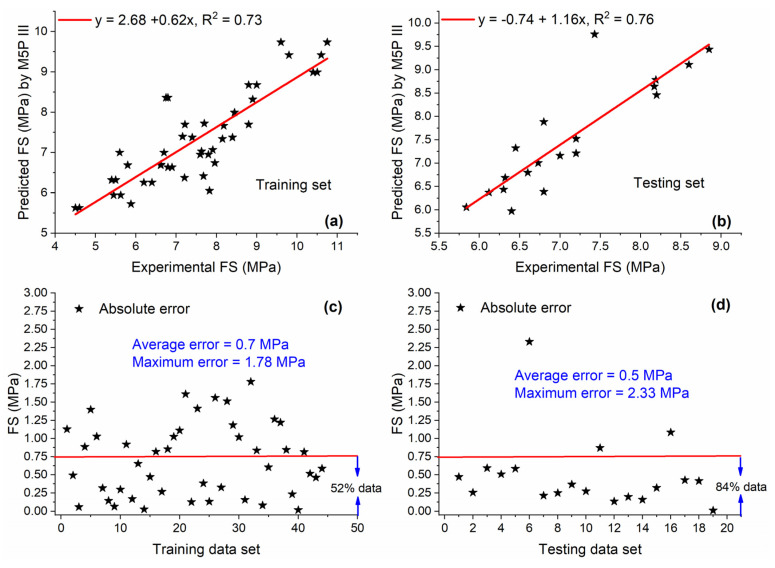
Experimental and predicted values of FS by M5P III for (**a**) training set and (**b**) testing set, and their corresponding absolute error for (**c**) training data set and (**d**) testing data set.

The prediction capability of M5P III is observed to be relatively poor as compared with GEP III and ANN III as indicated by Figure 21a,b which shows that R^2^ values for training and testing sets are 0.73 and 0.76, respectively. For the training set, this value was 17% and 23.16% lower as compared with GEP III and ANN III, respectively, while for the testing set, this value was 11.62% and 20% lower as compared with GEP III and ANN III, respectively. In addition, the relatively low performance of M5P III can be observed in Figure 21c, which shows that 48% of the data are greater than the AE of 0.75 MPa.

#### 5.3.4. RF III

**Figure 22:**
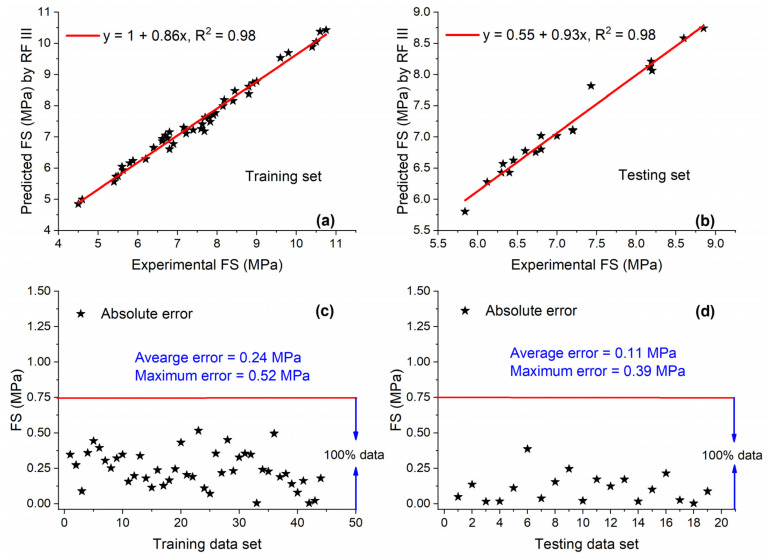
Experimental and predicted values of FS by RF III for (**a**) training set and (**b**) testing set, and their corresponding absolute error for (**c**) training data set and (**d**) testing data set.

As compared with other ML techniques for modelling FS of concrete with MK, excellent performance was observed by RF III. Figure 22a,b show an excellent correlation between predicted and experimental values, i.e., for both training and testing sets, the value of R^2^ = 0.98. This shows that RF III is a highly accurate, reliable, precise, and robust model. Figure 22c,d further strengthen the high accuracy of RF III, which shows that 100% of data are below 0.75 MPa for both data sets with low average errors.

#### 5.3.5. Comparison of GEP III, ANN III, M5P III, and RF III

**Figure 23:**
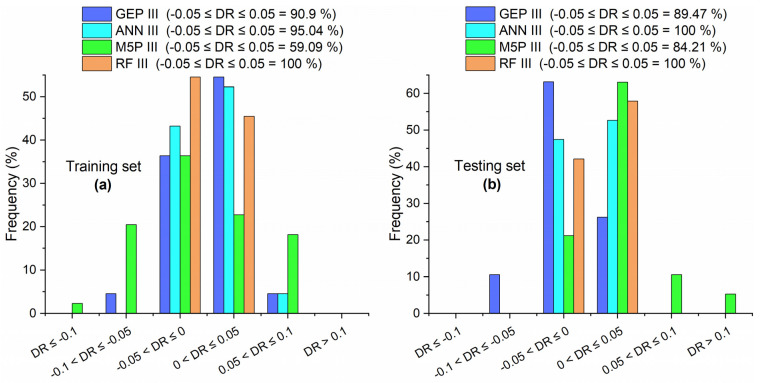
DR values of GEP III, ANN III, M5P III, and RF III for (**a**) training set and (**b**) testing set.

For both training and testing sets, the order of R between predicted and measured values by different models is RF III > ANN III > GEP III > M5P III, as shown in Table 7. The same order was observed by the value of R^2^ as discussed previously and by the percentage of DR that fall in the range of −0.05 to 0.05, as shown in Figure 23. In the case of the value of RMSE, RRMSE, RSE, and ρ, the order of models was RF III < ANN III < GEP III < M5P III for both data sets.

## 6. Sensitivity and Parametric Analysis

In order to find out the relative contribution of input parameters on outputs, sensitivity analysis (SA) was conducted by using the model proposed by Gandomi et al. [96] and as given in Equations (16) and (17).

(16) $$N_{i}=f_{max}\left(x_{i}\right)-f_{min}\left(x_{i}\right)$$

(17) $$S_{i}=\frac{N_{i}}{\sum_{j=1}^{n}N_{j}}\times 100$$

where, $f_{min}\left(x_{i}\right)$ and $f_{max}\left(x_{i}\right)$ are the minimum and maximum predicted outputs based on ith input variable, in which other input variables are kept constant at their mean values.

In order to calculate the variation in ${f^{\prime }}_{c}$, $f_{\mathrm{st}}$, and FS by changing MK content and days, parametric analysis (PA) was carried out. The PA was performed by observing a change in ${f^{\prime }}_{c}$, $f_{\mathrm{st}}$, and FS by increasing MK content and age of specimen from its minimum to maximum value while keeping all other input parameters at their mean values. The comparative study showed that RF performed better than the other modelling techniques; however, in this study, both sensitivity and parametric analysis was carried out by using GEP due to its convenience [79]. It is important to note that the accuracy of GEP models for the prediction of all three mechanical properties was high; therefore, it can be used to explore materials characteristics through sensitivity and parametric analysis.

**Figure 24:**
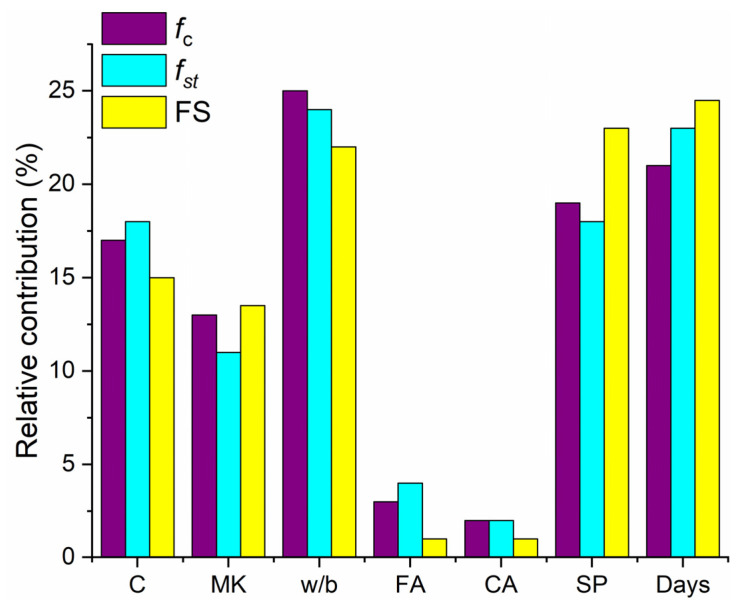
Relative contribution of input parameters to ${f^{\prime }}_{c}$, $f_{\mathrm{st}}$ and FS.

For both ${f^{\prime }}_{c}$ and $f_{\mathrm{st}}$, the w/b ratio seemed to be the most influential parameter, followed by days, SP, C, MK, FA, and CA, as shown in Figure 24. However, in the case of FS, the number of days is the most influential parameter, followed by SP, w/b ratio, C, MK, FA, and CA.

**Figure 25:**
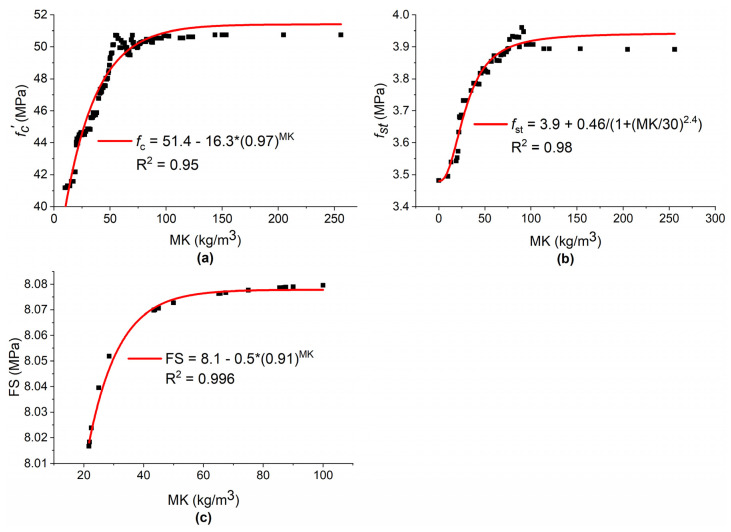
Variation in mechanical properties of concrete with MK: (**a**) ${f^{\prime }}_{c};$ (**b**) $f_{\mathrm{st}};$ (**c**) FS.

The PA in Figure 25 shows the variation in mechanical properties of concrete by increasing MK content from its minimum to maximum value. The development of concrete strength with MK incorporation can be attributed to: (i) pozzolanic reaction of MK with calcium hydroxide, (ii) acceleration of cement hydration, and (iii) the filling effect due to MK particles [15]. In mortar with MK, the formation of alumina phases such as C_2_ASH_8_ is responsible for higher strength at early ages [97]. The rapid and early pozzolanic reaction of MK with CH may decrease the initial and final setting times of concrete with MK [55]. Figure 25a depicts that for up to 35 kg/m^3^ addition of MK (which is about 10% cement replacement), ${f^{\prime }}_{c}$ increases linearly. After further increasing MK content up to 69 kg/m^3^ (about 15% cement replacement), the ${f^{\prime }}_{c}$ increases but nonlinearly. MK content from 69 to 105 kg/m^3^ (about 25% cement placement) increases the ${f^{\prime }}_{c}$ nonlinearly but with slower rate as compared with the 69 kg/m^3^. Further increase in MK beyond 105 kg/m^3^ does not increase ${f^{\prime }}_{c}$ significantly. Similar to our results, Rahmat et al. [15] investigated ${f^{\prime }}_{c}$ of different SCC with MK incorporation (0–20%) at different w/b ratios and at different curing ages and observed that the most remarkable strength developments were found with 10–15% MK replacement. Hamdy et al. [39] investigated six different proportions of 0, 10, 15, 20, 30, 40, and 50% cement replacement with MK for high-strength concrete and observed ${f^{\prime }}_{c}$ at 3 and 7 days. They concluded that maximum ${f^{\prime }}_{c}$ was observed at 15% MK replacement. After the 30%, the ${f^{\prime }}_{c}$ was observed to be lowered as compared with plain concrete. The decrease in ${f^{\prime }}_{c}$ by increasing MK content beyond optimum content may be due to the reason that higher MK content decreases the CaO/SiO_2_ ratio [72], which results in the higher requirement of SP [34] and clinker dilution effect due to partial replacement of cement with MK [46]. Moreover, at a low w/c ratio, increasing MK% beyond 15% decreased the ${f^{\prime }}_{c}$ and $f_{\mathrm{st}}$, as, in this case, less calcium hydroxide is available for reaction with MK [39]. Figure 25b shows that from 10–40 kg/m^3^ MK content (about 2.5–10% cement replacement), $f_{\mathrm{st}}$ increases almost linearly. By further increasing MK content up to 60 kg/m^3^ (about 15% cement replacement), $f_{\mathrm{st}}$ increases non-linearly, but with a faster rate as compared with further increase in MK content. Above 100 kg/m^3^ (or about 25% cement replacement), no significant improvement in $f_{\mathrm{st}}$ is observed. Rahmat et al. [15] also observed that SCC showed better $f_{\mathrm{st}}$ with 10–15% MK as cement replacement. Kannan et al. [53] incorporated five different proportions of 5, 10, 15, 20, 25, and 30% MK as particle cement replacement and observed a maximum value of $f_{\mathrm{st}}$ at 20% and then a decrease in $f_{\mathrm{st}}$ beyond this percentage. The trend of $f_{\mathrm{st}}$ with MK was similar to that observed for ${f^{\prime }}_{c}$ with MK. Figure 25c depicts that from the incorporation of MK up to 50 kg/m^3^ (about 10% cement replacement), FS increases significantly. From 50 to 100 kg/m^3^ MK content (about 10 to 20% cement replacement), the increase in FS was not significant. Lenka et al. [4] also observed that concrete gave a maximum performance in terms of FS at 10% MK inclusion as cement replacement.

**Figure 26:**
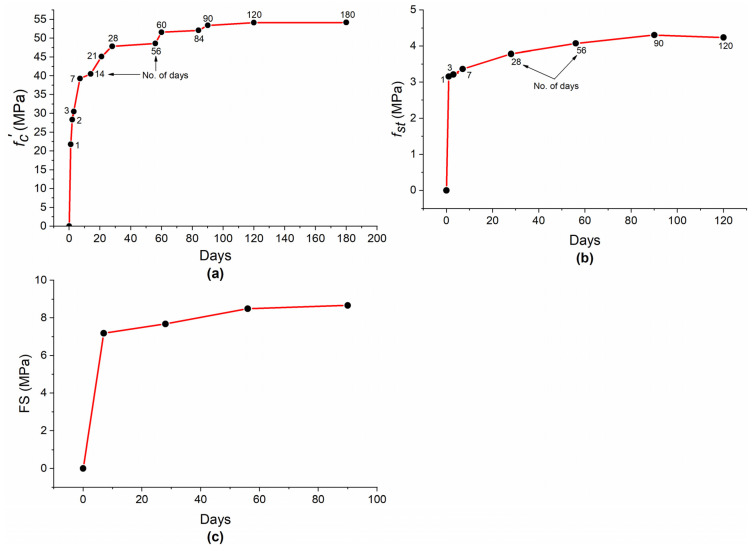
Variation in mechanical properties of concrete with age: (**a**)${f^{\prime }}_{c};$ (**b**)$f_{\mathrm{st}};$ (**c**) FS.

Figure 26a shows that the rate of strength development at early ages (up to 7 days) is fast, which can be attributed to the fast pozzolanic reaction of MK [98]. Bai et al. [99] observed an up to 92% increase in early age strength with the incorporation of 5% MK as compared with plain concrete. Erhan et al. [16] found that concrete strength with MK at early ages (1–7 days) was 5–23% greater as compared with plain concrete, depending on MK replacement level and w/b ratio. From 14–60 days, the ${f^{\prime }}_{c}$ development of concrete with MK was slow and no significant strength development was observed after 90 days. Figure 26b shows that, similar to ${f^{\prime }}_{c}$, the development of $f_{\mathrm{st}}$ at an early age (1 day) is very high as compared with further increase in time. From 3 to 90 days, $f_{\mathrm{st}}$ increased but with a much slower rate as compared with day 1. Similar to ${f^{\prime }}_{c}$ and $f_{\mathrm{st}}$, the rate of FS development is higher in the first 7 days as compared with the rest of the days, as shown in Figure 26c.

## 7. Conclusions

Compressive strength (${f^{\prime }}_{c}$), splitting tensile strength ($f_{\mathrm{st}}$), and flexural strength (FS) of concrete are the parameters of the design in many codes. An accurate and reliable estimation of these parameters can save cost and time, and help in scheduling activities such as formwork removal. In this study, ${f^{\prime }}_{c}$, $f_{\mathrm{st}}$, and FS of concrete with the incorporation of metakaolin (MK) as partial cement replacement were modelled using four machine learning (ML) techniques: gene expression programming (GEP), artificial neural network (ANN), M5P model tree algorithm, and random forest (RF). For this purpose, a comprehensive database was gathered from peer-reviewed published documents. The database used in the modelling was comprised of 982 data points for ${f^{\prime }}_{c}$, 204 data points for $f_{\mathrm{st}}$, and 63 data samples for FS of concrete with MK. For all three databases, the input parameters were cement, MK, w/b, fine and coarse aggregates, superplasticizer, and age of a specimen in days. Many statistical metrics were used to compare the predictive performance of ML techniques used in this study. In the end, sensitivity and parametric analysis (PA) was performed. Based on the application of GEP, ANN, M5P, and RF for predicting ${f^{\prime }}_{c}$, $f_{\mathrm{st}}$, and FS of concrete with MK, the following conclusions can be drawn:

1. For modelling ${f^{\prime }}_{c}$ of concrete with MK, RF I (R^2^ = 0.99) showed excellent predictive capability followed by ANN I (R^2^ = 0.94) and GEP I (R^2^ = 0.81) for both training and testing sets. These results were also supported by other statistical metrics such as R, RMSE, RSE, MAE, DR, and ρ.
2. For the training set, in the case of the $f_{\mathrm{st}}$ prediction, RF II performed better with R^2^ = 0.98 followed by ANN II (R^2^ = 0.92), M5P II (R^2^ = 0.88), and GEP II (R^2^ = 0.86). A slight change was observed in the order of ML techniques in the case of the testing set, where GEP II (R^2^ = 0.90) performed well as compared with M5P II (R^2^ = 0.86), while the order of RF II and ANN III was the same as observed for the training set.
3. Similar to the prediction results of ${f^{\prime }}_{c}$ and $f_{\mathrm{st}}$ database, RF III remained on top with respect to its excellent prediction performance as compared with other ML techniques for the FS database. The values of R^2^ equal to 0.98 and 0.98 were observed by RF III and ANN III for both training and testing sets. For the FS database, M5P III’s performance was relatively low as compared with other ML techniques and showed R^2^ = 0.73 and 0.76 for training and testing sets, respectively. GEP III showed better prediction potential as compared with M5P III with R^2^ = 0.88 and 0.86 for training and testing sets, respectively.
4. PA analysis showed that 15% MK incorporation as partial cement replacement was suitable for both ${f^{\prime }}_{c}$ and $f_{\mathrm{st}}$, while this content was 10% for FS. In addition, significant strength development was observed at early ages with MK incorporation for all the mechanical properties.

## 8. Future Research

1. In this study, four individual machine learning techniques were used for predicting the mechanical properties of concrete with MK. It would be beneficial to use the ensemble ML technique and compare it with individual ML techniques.
2. More properties of concrete with MK such as rheology, elastic modulus, and durability characteristics need to be modelled by using advanced ML techniques.

## Figures and Tables

**Table 1:** Descriptive statistics of input and output variables used in the training set.

Statistical Indicator	C (kg/m^3^)	MK (kg/m^3^)	w/b Ratio	FA (kg/m^3^)	CA (kg/m^3^)	SP (kg/m^3^)	Days	Strength (MPa)
	${f^{\prime }}_{c}$ Database							
Minimum	176.25	0	0.21	272.5	0	0	1	4
Maximum	680	256	0.8	1502	1510	24	180	107
Mean	384.77	44.35	0.447	765	991	3.6	36	48.86
Standard error	2.8	1.15	0.004	5.95	8.88	0.125	1.4	0.73
Standard deviation	87	36.26	0.124	186.3	278.33	3.91	44.54	22.85
Kurtosis	−0.13	3.59	0.45	3.29	2.3	7.44	3.83	−0.435
Skewness	0.03	1.1	0.73	1.14	−1.3	2.16	2.07	0.48
	$f_{\mathrm{st}}$ **Database**							
Minimum	266	0	0.21	272.5	175.1	0	1	1.1
Maximum	570	256	0.75	989	1265	12.4	120	5.88
Mean	400	44.1	0.44	756	866	4.23	34.62	3.44
Standard error	4.59	2.72	0.008	12.63	18.64	0.23	2.21	0.071
Standard deviation	65.69	39	0.12	180.83	267	3.34	31.67	1.01
Kurtosis	−0.36	4.2	−0.005	−0.39	1.6	−0.68	0.37	−0.25
Skewness	0.14	1.31	0.41	−0.58	−1.11	0.41	1.23	0.42
	**FS Database**							
Minimum	304	0	0.28	624.8	822	0	7	4.5
Maximum	570	100	0.48	843	1265	8.55	90	10.75
Mean	399.5	44.22	0.415	716	1051	1.97	39.98	7.38
Standard error	7.21	4.04	0.006	11.44	20.7	0.24	3.89	0.18
Standard deviation	57.21	32.05	0.051	90.83	164.5	1.94	30.89	1.42
Kurtosis	0.59	−1.31	1.024	−1.61	−1.3	0.92	−0.95	0.055
Skewness	0.31	0.03	−1.085	0.5	−0.22	1.01	0.74	0.461

**Table 2:** Parameters of developed GEP models.

Parameters	GEP I	GEP II	GEP III
Genes	4	5	3
Head size	13	10	8
Chromosomes	50	30	250
Function set	+, −, ∗, /, Sqrt, Exp, Ln, Inv, X2, X3, X4, X5, 4Rt, 5Rt, Sin, Cos, Tan, Sec, Cosh, Tanh, Coth, Sech	+, −, ∗, /, Sqrt, Exp, Ln, Log, Inv, 3Rt, Cos, Tan, Cot, Sec, Coth, Tanh, Sech	+, −, ∗, /
Linking function	Multiplication	Addition	Addition
Generation	400,000	70,000	50,000
Fitness function error type	RMSE	RMSE	RMSE
Mutation rate	0.00138	0.00138	0.00138

**Table 3:** Different parameters of the GEP algorithm used by researchers to obtain a reliable and robust model.

No. of Chromosomes	Head Size	No. of Genes	Linking Function	Function Set	Output	R^2^ (Training Set)	R^2^ (Testing Set)	Ref.
30	10	4	Addition	+, −, ∗, /, X2, 3Rt	${f^{\prime }}_{c}$ of concrete with bagasse ash	0.83	0.85	[21]
30	10	4	Addition	+, −, ∗, /	${f^{\prime }}_{c}$ of high strength concrete	0.91	0.9	[25]
26	12	3	Multiplication	+, −, ∗, /, Sqrt, X3	${f^{\prime }}_{c}$ of geopolymer concrete with blast-furnace slag	0.92	0.94	[91]
20	4	2	Multiplication	+, −, ∗, /, Sqrt	$f_{\mathrm{st}}$$\;\mathrm{by}\;\mathrm{using}\;{f^{\prime }}_{c}$ and w/b	0.87	0.88	[92]

**Table 4:** Statistical evaluation of GEP I, ANN I, and RF I.

Model	Training Set					Testing Set				
	R	RMSE	RRMSE	RSE	ρ	R	RMSE	RRMSE	RSE	ρ
GEP I	0.9	9.3	0.19	0.19	0.1	0.9	9.43	0.2	0.19	0.12
ANN I	0.97	5.49	0.12	0.063	0.061	0.97	5.18	0.1	0.063	0.051
RF I	0.997	2.03	0.044	0.01	0.02	0.996	1.86	0.04	0.01	0.02

**Table 5:** Coefficients of linear models developed by M5P II based on Equation (3).

Models	Coefficients							
	a	b	c	d	e	f	g	h
LM 1	5.52	0	−0.0002	−1.19	−0.003	0	0.145	0.03
LM 2	7.06	−0.003	0.005	−6.091	−0.001	0.0002	0.015	0.0214
LM 3	5.2	−0.003	0.0037	−5.55	0.0017	0.0002	0.0151	0.0151
LM 4	5.78	−0.005	0.0035	−4.62	−0.0002	0.0015	0.0151	0.022
LM 5	8.08	−0.0041	0.0015	−8.45	0.0006	0.0003	0.063	0.01
LM 6	8.4	−0.004	0.0015	−8.45	0.0003	0.0003	0.074	0.01
LM 7	3.732	0.0016	0.004	−2.3	−0.0006	0	−0.0053	0.011
LM 8	9.6	0.0025	0.0076	−2.955	−0.0077	0	−0.049	0.0574
LM 9	6.9	0.0047	0.0099	−2.0359	−0.0057	0	−0.0404	0.0063
LM 10	15.8	0.0013	0.012	−5.56	−0.012	0	−0.047	0.005
LM 11	15.8	0.0013	0.0119	−5.56	−0.012	0	−0.047	0.005
LM 12	10.57	0.0032	0.011	−4.43	−0.008	0	−0.047	0.005

**Table 6:** Statistical evaluation of GEP II, ANN II, M5P II, and RF II.

Model	Training Set					Testing Set				
	R	RMSE	RRMSE	RSE	ρ	R	RMSE	RRMSE	RSE	ρ
GEP II	0.93	0.378	0.111	0.14	0.06	0.95	0.339	0.096	0.11	0.05
ANN II	0.96	0.2816	0.0836	0.08	0.043	0.98	0.198	0.0548	0.04	0.03
M5P II	0.94	0.3547	0.1053	0.12	0.05	0.93	0.4053	0.112	0.17	0.06
RF II	0.99	0.135	0.04	0.02	0.02	0.99	0.122	0.0337	0.015	0.02

**Table 7:** Statistical evaluation of GEP III, ANN III, M5P III, and RF III.

Model	Training Set					Testing Set				
	R	RMSE	RRMSE	RSE	ρ	R	RMSE	RRMSE	RSE	ρ
GEP III	0.94	0.5326	0.07226	0.125	0.04	0.93	0.455	0.0616	0.16	0.03
ANN III	0.98	0.3522	0.048	0.055	0.02	0.97	0.2753	0.0373	0.06	0.02
M5P III	0.85	0.858	0.1146	0.3	0.06	0.87	0.7054	0.099	0.66	0.05
RF III	0.99	0.247	0.0366	0.03	0.018	0.99	0.147	0.0207	0.03	0.01

## Data Availability

The data presented in this study are available in the Supplementary Material.

## Supplementary Materials

The following supporting information can be downloaded at: https://www.mdpi.com/article/10.3390/ma15155435/s1, Table S1: Experimental database of ${f^{\prime }}_{c}$ of concrete with metakaolin. Table S2: Experimental database of $f_{\mathrm{st}}$ of concrete with metakaolin. Table S3: Experimental database of FS of concrete with metakaolin. References [4,15,16,17,18,33,34,35,36,37,38,39,40,41,42,43,44,45,46,47,48,49,50,51,52,53,54,55,56,57,58,59,60,61,62,63,64,65,66,67,68,69,70,72,73] are cited in the supplementary materials.
