# Supplementary material for: Application of Machine Learning Techniques for Predicting Compressive, Splitting Tensile, and Flexural Strengths of Concrete with Metakaolin
Source: Materials (Basel). 2022 Aug 7;15(15):5435. doi: 10.3390/ma15155435 (PMC9369826; doi:10.3390/ma15155435)
Supplement: Supplementary file 1 [file materials-15-05435-s001.zip › materials-1818162-supplementary.pdf]

Application of machine learning techniques for predicting compressive, splitting tensile, and flexural strength of concrete with metakaolin

Supplementary Information

Table S1: Experimental database of f'_c of concrete with metakaolin.

S.No.	Cement (kg/m^3)	Metakaolin (kg/m^3)	w/b	Fine aggregate (kg/m^3)	Coarse aggregate (kg/m^3)	SP (kg/m^3)	Days	f'_c (MPa)	Geometry of Specimen (mm)	Ref.
1	335.4	0	0.4	670.8	1341.2	0	28	19.5	cube 100	[1]
2	251.47	62.87	0.599987	628.68	1257.4	0	28	13		
3	259.6	64.9	0.5	649	1298	0	28	14.7		
4	267.17	47.15	0.600025	628.68	1257.4	0	28	15		
5	268.32	67.08	0.4	670.8	1341.2	0	28	17		
6	275.83	48.68	0.499985	649	1298	0	28	16		
7	282.91	31.43	0.599987	628.68	1257.4	0	28	19		
8	285.09	50.31	0.4	670.8	1341.2	0	28	20		
9	292.05	32.45	0.5	649	1298	0	28	20		
10	298.62	15.72	0.599987	628.68	1257.4	0	28	17.7		
11	301.86	33.54	0.4	670.8	1341.2	0	28	21		
12	305.9	76.5	0.599895	573.5	1147.02	0	28	16		
13	308.28	16.23	0.499985	649	1298	0	28	19.8		
14	314.34	0	0.599987	628.68	1257.4	0	28	17		
15	318.08	79.52	0.5	596.4	1192.8	0	28	16.3		
16	318.63	16.77	0.4	670.8	1341.2	0	28	20.2		
17	324.5	0	0.5	649	1298	0	28	18		
18	325	57.35	0.599974	573.5	1147.02	0	28	18.41		
19	331.2	82.8	0.4	621	1242	0	28	14.5		
20	337.96	59.64	0.5	596.4	1192.8	0	28	18.5		
21	344.12	38.32	0.599833	573.5	1147.02	0	28	25.53		
22	351.9	62.1	0.4	621	1242	0	28	19		
23	357.84	39.76	0.5	596.4	1192.8	0	28	25		
24	363.2	19.12	0.600021	573.5	1147.02	0	28	24		
25	372.6	41.4	0.4	621	1242	0	28	25.66		
26	377.7	19.88	0.500025	596.4	1192.8	0	28	23.65		
27	382.34	0	0.59999	573.5	1147.02	0	28	23.43		
28	393.3	20.7	0.4	621	1242	0	28	24.2		
29	397.6	0	0.5	596.4	1192.8	0	28	22.22		
30	414	0	0.4	621	1242	0	28	23.4		
31	426	0	0.380282	720	1080	4.26	28	38.5	cylinder 100*200	[2]
32	362	64	0.380282	720	1080	4.26	28	69.2		
33	383	43	0.380282	720	1080	4.26	28	63.6		

34	405	21	0.380282	720	1080	4.26	28	45.9		
35	533.33	0	0.300002	677	1201	2.13	3	56.4	cube 100	[3]
36	453.33	80	0.300002	645	1144	4.26	3	48.93		
37	453.33	80	0.300002	645	1144	4.26	7	79.88		
38	453.33	80	0.300002	645	1144	4.26	28	91.04		
39	453.33	80	0.300002	645	1144	4.26	90	102.96		
40	480	53.33	0.300002	655	1163	3.47	3	53.96		
41	480	53.33	0.300002	655	1163	3.47	7	77.85		
42	480	53.33	0.300002	655	1163	3.47	28	98.81		
43	480	53.33	0.300002	655	1163	3.47	90	106.13		
44	506.67	26.67	0.299996	666	1182	2.93	3	59.45		
45	506.67	26.67	0.299996	666	1182	2.93	7	78.74		
46	506.67	26.67	0.299996	666	1182	2.93	28	95.6		
47	506.67	26.67	0.299996	666	1182	2.93	90	102.5		
48	533.33	0	0.300002	677	1201	2.13	7	78.23		
49	533.33	0	0.300002	677	1201	2.13	28	91.87		
50	533.33	0	0.300002	677	1201	2.13	90	101		
51	500	0	0.3	725	1087	0.5	3	68.5	cube 100	[4]
52	328	82	0.5	653	1081	0	3	30		
53	328	82	0.5	653	1081	0	7	43.2		
54	328	82	0.5	653	1081	0	28	58.4		
55	328	82	0.5	653	1081	0	90	69.1		
56	369	41	0.5	656	1081	0	3	40.4		
57	369	41	0.5	656	1081	0	7	55.2		
58	369	41	0.5	656	1081	0	28	66.2		
59	369	41	0.5	656	1081	0	90	71.6		
60	390	20.5	0.5	659	1081	0	3	32.6		
61	390	20.5	0.5	659	1081	0	7	45.9		
62	390	20.5	0.5	659	1081	0	28	57.1		
63	390	20.5	0.5	659	1081	0	90	66.5		
64	400	100	0.3	708	1087	1	3	70.8		
65	400	100	0.3	708	1087	1	7	87.6		
66	400	100	0.3	708	1087	1	28	99.6		
67	410	0	0.5	662	1081	0	3	28.6		
68	410	0	0.5	662	1081	0	7	41.2		
69	410	0	0.5	662	1081	0	28	52.1		
70	410	0	0.5	662	1081	0	90	60.4		
71	450	50	0.3	717	1087	0.8	3	85.9		
72	450	50	0.3	717	1087	0.8	7	99.8		
73	475	25	0.3	721	1087	0.6	3	73		
74	475	25	0.3	721	1087	0.6	7	88.8		
75	475	25	0.3	721	1087	0.6	28	103.6		
76	500	0	0.3	725	1087	0.5	7	81.1		

77	500	0	0.3	725	1087	0.5	28	96.5		
78	500	0	0.3	725	1087	0.5	90	102.5		
79	490	0	0.32	967	817	2.47	3	19.5	cube 100	[5]
80	348	87	0.45	945	785	3.91	3	13.8		
81	348	87	0.45	945	785	3.91	7	22		
82	348	87	0.45	945	785	3.91	14	30		
83	348	87	0.45	945	785	3.91	28	35.6		
84	348	87	0.45	945	785	3.91	56	43.2		
85	368	92	0.38	970	781	4.89	3	19		
86	368	92	0.38	970	781	4.89	7	32		
87	368	92	0.38	970	781	4.89	14	41.4		
88	368	92	0.38	970	781	4.89	28	46		
89	368	92	0.38	970	781	4.89	56	53.2		
90	370	65.2	0.45	940	795	3.26	3	14.6		
91	370	65.2	0.45	940	795	3.26	7	23.6		
92	370	65.2	0.45	940	795	3.26	14	32.5		
93	370	65.2	0.45	940	795	3.26	28	38.3		
94	370	65.2	0.45	940	795	3.26	56	45.4		
95	391	69	0.38	964	790	4.89	3	19.9		
96	391	69	0.38	964	790	4.89	7	34.6		
97	391	69	0.38	964	790	4.89	14	43.1		
98	391	69	0.38	964	790	4.89	28	49.1		
99	391	69	0.38	964	790	4.89	56	54.5		
100	392	98	0.32	985	795	9.96	3	20		
101	392	43.5	0.45	933	800	2.93	3	15.5		
102	392	98	0.32	985	795	9.96	7	36		
103	392	43.5	0.45	933	800	2.93	7	24		
104	392	98	0.32	985	795	9.96	14	42		
105	392	43.5	0.45	933	800	2.93	14	31.8		
106	392	98	0.32	985	795	9.96	28	47		
107	392	43.5	0.45	933	800	2.93	28	36.8		
108	392	98	0.32	985	795	9.96	56	54.9		
109	392	43.5	0.45	933	800	2.93	56	46.5		
110	414	46	0.38	958	800	3.6	3	20		
111	414	21.8	0.45	925	805	1.95	3	13.9		
112	414	46	0.38	958	800	3.6	7	33.3		
113	414	21.8	0.45	925	805	1.95	7	22.5		
114	414	46	0.38	958	800	3.6	14	42.3		
115	414	21.8	0.45	925	805	1.95	14	30.4		
116	414	46	0.38	958	800	3.6	28	49.5		
117	414	21.8	0.45	925	805	1.95	28	34		
118	414	46	0.38	958	800	3.6	56	53.9		
119	414	21.8	0.45	925	805	1.95	56	42.4		

120	417	73.5	0.32	980	800	7.35	3	22		
121	417	73.5	0.32	980	800	7.35	7	38.7		
122	417	73.5	0.32	980	800	7.35	14	44.5		
123	417	73.5	0.32	980	800	7.35	28	51.4		
124	417	73.5	0.32	980	800	7.35	56	59.3		
125	435	0	0.45	920	815	1.63	3	13		
126	435	0	0.45	920	815	1.63	7	19		
127	435	0	0.45	920	815	1.63	14	26.3		
128	435	0	0.45	920	815	1.63	28	31.2		
129	435	0	0.45	920	815	1.63	56	39.9		
130	437	23	0.38	947	810	3.31	3	19.3		
131	437	23	0.38	947	810	3.31	7	32		
132	437	23	0.38	947	810	3.31	14	40.5		
133	437	23	0.38	947	810	3.31	28	45.7		
134	437	23	0.38	947	810	3.31	56	51.6		
135	441	49	0.32	975	805	7.02	3	23.5		
136	441	49	0.32	975	805	7.02	7	40		
137	441	49	0.32	975	805	7.02	14	45.8		
138	441	49	0.32	975	805	7.02	28	53.6		
139	441	49	0.32	975	805	7.02	56	60		
140	460	0	0.38	940	815	1.73	3	18		
141	460	0	0.38	940	815	1.73	7	30.3		
142	460	0	0.38	940	815	1.73	14	35.2		
143	460	0	0.38	940	815	1.73	28	41.9		
144	460	0	0.38	940	815	1.73	56	45.9		
145	466	24.5	0.32	967	815	4.57	3	20		
146	466	24.5	0.32	967	815	4.57	7	36		
147	466	24.5	0.32	967	815	4.57	14	43		
148	466	24.5	0.32	967	815	4.57	28	49.2		
149	466	24.5	0.32	967	815	4.57	56	58		
150	490	0	0.32	967	817	2.47	7	33.8		
151	490	0	0.32	967	817	2.47	14	37		
152	490	0	0.32	967	817	2.47	28	45		
153	490	0	0.32	967	817	2.47	56	53.2		
154	400	0	0.5	935	765	0	7	19	cube 100	[6]
155	340	60	0.5	935	765	0	7	25.1		
156	340	60	0.5	935	765	0	28	40		
157	340	60	0.5	935	765	0	90	48		
158	340	60	0.5	935	765	0	180	51.5		
159	350	50	0.5	935	765	0	7	23		
160	350	50	0.5	935	765	0	28	40		
161	350	50	0.5	935	765	0	90	47.2		
162	350	50	0.5	935	765	0	180	50.5		

163	360	40	0.5	935	765	0	7	26		
164	360	40	0.5	935	765	0	28	41		
165	360	40	0.5	935	765	0	90	46		
166	360	40	0.5	935	765	0	180	50		
167	400	0	0.5	935	765	0	28	35.5		
168	400	0	0.5	935	765	0	90	43		
169	400	0	0.5	935	765	0	180	48.5		
170	450	0	0.35	957	852	7.9	1	29	cube 100	[7]
171	280	70	0.55	953	848	6.1	1	15		
172	280	70	0.55	953	848	6.1	3	30		
173	280	70	0.55	953	848	6.1	7	39.25		
174	280	70	0.55	953	848	6.1	28	52.5		
175	280	70	0.55	953	848	6.1	90	61.5		
176	280	70	0.55	953	848	6.1	120	70		
177	315	35	0.55	956	851	4.4	1	13		
178	315	35	0.55	956	851	4.4	3	26.5		
179	315	35	0.55	956	851	4.4	7	36.5		
180	315	35	0.55	956	851	4.4	28	50		
181	315	35	0.55	956	851	4.4	90	59		
182	315	35	0.55	956	851	4.4	120	66.5		
183	350	0	0.55	960	854	3.5	1	13		
184	350	0	0.55	960	854	3.5	3	24.5		
185	350	0	0.55	960	854	3.5	7	33		
186	350	0	0.55	960	854	3.5	28	46		
187	350	0	0.55	960	854	3.5	90	50.05		
188	350	0	0.55	960	854	3.5	120	54.5		
189	360	90	0.35	948	844	12.4	1	31		
190	360	90	0.35	948	844	12.4	3	52		
191	360	90	0.35	948	844	12.4	7	67		
192	360	90	0.35	948	844	12.4	28	78.5		
193	360	90	0.35	948	844	12.4	90	93		
194	360	90	0.35	948	844	12.4	120	98.5		
195	405	45	0.35	952	848	10.1	1	33		
196	405	45	0.35	952	848	10.1	3	48		
197	405	45	0.35	952	848	10.1	7	58		
198	405	45	0.35	952	848	10.1	28	75		
199	405	45	0.35	952	848	10.1	90	85.5		
200	405	45	0.35	952	848	10.1	120	92		
201	450	0	0.35	957	852	7.9	3	50		
202	450	0	0.35	957	852	7.9	7	54		
203	450	0	0.35	957	852	7.9	28	69		
204	450	0	0.35	957	852	7.9	90	72		
205	450	0	0.35	957	852	7.9	120	77		

206	437.78	0	0.449998	643.68	1104.36	0	7	28.74	cube 150	[8]
207	350.22	87.56	0.449998	643.68	1104.36	0	7	38.96		
208	350.22	87.56	0.449998	643.68	1104.36	0	28	45.49		
209	372.11	65.67	0.449998	643.68	1104.36	0	7	41.19		
210	372.11	65.67	0.449998	643.68	1104.36	0	28	51.56		
211	394	43.78	0.449998	643.68	1104.36	0	7	38.07		
212	394	43.78	0.449998	643.68	1104.36	0	28	43.7		
213	415.89	21.89	0.449998	643.68	1104.36	0	7	32.07		
214	415.89	21.89	0.449998	643.68	1104.36	0	28	38.22		
215	437.78	0	0.449998	643.68	1104.36	0	28	37.04		
216	482	0	0.30083	608	1254	6.5552	1	28.9	cube 100	[9]
217	447	96	0.309392	705	1454	7.3848	1	22.8		
218	447	96	0.309392	705	1454	7.3848	7	62.8		
219	447	96	0.309392	705	1454	7.3848	14	78.9		
220	447	96	0.309392	705	1454	7.3848	28	83.2		
221	447	96	0.309392	705	1454	7.3848	56	86.1		
222	457	72	0.304348	678	1398	7.1944	1	28.6		
223	457	72	0.304348	678	1398	7.1944	7	70.2		
224	457	72	0.304348	678	1398	7.1944	14	83.6		
225	457	72	0.304348	678	1398	7.1944	28	89.7		
226	457	72	0.304348	678	1398	7.1944	56	91		
227	462	60	0.302682	665	1372	7.0992	1	28.5		
228	462	60	0.302682	665	1372	7.0992	7	68.6		
229	462	60	0.302682	665	1372	7.0992	14	81.6		
230	462	60	0.302682	665	1372	7.0992	28	85.3		
231	462	60	0.302682	665	1372	7.0992	56	90.4		
232	470	36	0.302372	641	1322	6.8816	1	25.1		
233	470	36	0.302372	641	1322	6.8816	7	62.2		
234	470	36	0.302372	641	1322	6.8816	14	74.4		
235	470	36	0.302372	641	1322	6.8816	28	81.4		
236	470	36	0.302372	641	1322	6.8816	56	85.7		
237	474	24	0.301205	629	1298	6.7728	1	30.6		
238	474	24	0.301205	629	1298	6.7728	7	59.2		
239	474	24	0.301205	629	1298	6.7728	14	67.7		
240	474	24	0.301205	629	1298	6.7728	28	72.5		
241	474	24	0.301205	629	1298	6.7728	56	79		
242	482	0	0.30083	608	1254	6.5552	7	55.3		
243	482	0	0.30083	608	1254	6.5552	14	60.7		
244	482	0	0.30083	608	1254	6.5552	28	66.1		
245	482	0	0.30083	608	1254	6.5552	56	71.3		
246	480	0	0.44	900	670	7.2	7	27.53	cube 150	[10]
247	408	72	0.44	900	670	8.64	7	38.1		
248	408	72	0.44	900	670	8.64	28	51.7		

249	432	48	0.44	900	670	8.64	7	35.8		
250	432	48	0.44	900	670	8.64	28	47.9		
251	456	24	0.44	900	670	8.64	7	32.6		
252	456	24	0.44	900	670	8.64	28	45.5		
253	480	0	0.44	900	670	7.2	28	41.4		
254	512	0	0.319141	594	1037	5.12	3	28.84	cube 100	[11]
255	512	51.2	0.290128	594	1037	5.12	3	29.18		
256	512	76.8	0.277514	594	1037	5.12	3	29.69		
257	512	102.4	0.265951	594	1037	5.12	3	29.01		
258	512	153.6	0.245493	594	1037	5.12	3	26.8		
259	512	204.8	0.227958	594	1037	5.12	3	25.96		
260	512	256	0.21276	594	1037	5.12	3	24.09		
261	512	0	0.319141	594	1037	5.12	7	46.48		
262	512	51.2	0.290128	594	1037	5.12	7	50.73		
263	512	76.8	0.277514	594	1037	5.12	7	53.61		
264	512	102.4	0.265951	594	1037	5.12	7	50.39		
265	512	153.6	0.245493	594	1037	5.12	7	48.25		
266	512	204.8	0.227958	594	1037	5.12	7	44.96		
267	512	256	0.21276	594	1037	5.12	7	42.41		
268	512	0	0.319141	594	1037	5.12	28	61.07		
269	512	51.2	0.290128	594	1037	5.12	28	70.74		
270	512	76.8	0.277514	594	1037	5.12	28	74.48		
271	512	102.4	0.265951	594	1037	5.12	28	69.73		
272	512	153.6	0.245493	594	1037	5.12	28	65.82		
273	512	204.8	0.227958	594	1037	5.12	28	62.26		
274	512	256	0.21276	594	1037	5.12	28	59.89		
275	400	0	0.5	545	1012	0	7	26	cube 150	[12]
276	400	10	0.554268	490.5	1012	0	7	27.5		
277	400	20	0.605952	436	1012	0	7	29.5		
278	400	30	0.655233	317.8	1012	0	7	31.25		
279	400	40	0.702273	327	1012	0	7	33.5		
280	400	50	0.747222	272.5	1012	0	7	25.5		
281	400	0	0.5	545	1012	0	28	30.5		
282	400	10	0.554268	490.5	1012	0	28	32.5		
283	400	20	0.605952	436	1012	0	28	35		
284	400	30	0.655233	317.8	1012	0	28	37.5		
285	400	40	0.702273	327	1012	0	28	40		
286	400	50	0.747222	272.5	1012	0	28	30		
287	400	0	0.5	545	1012	0	91	38		
288	400	10	0.554268	490.5	1012	0	91	39		
289	400	20	0.605952	436	1012	0	91	41		
290	400	30	0.655233	317.8	1012	0	91	42.5		
291	400	40	0.702273	327	1012	0	91	47		

292	400	50	0.747222	272.5	1012	0	91	34.5		
293	450	0	0.45	482	1040	4.95	35	33	cylinder 100*200	[13]
294	383	68	0.45	482	1040	4.95	35	32		
295	383	68	0.45	482	1040	4.95	56	30.25		
296	383	68	0.45	482	1040	4.95	84	34.25		
297	405	45	0.45	482	1040	4.95	35	29.25		
298	405	45	0.45	482	1040	4.95	56	34.75		
299	405	45	0.45	482	1040	4.95	84	39.25		
300	428	23	0.45	482	1040	4.95	35	34		
301	428	23	0.45	482	1040	4.95	56	36.5		
302	428	23	0.45	482	1040	4.95	84	42		
303	450	0	0.45	482	1040	4.95	56	38		
304	450	0	0.45	482	1040	4.95	84	43		
305	400	0	0.4	714	1165	2.4	3	27	cube 100	[14]
306	364	36	0.4	714	1165	2.4	3	28.3		
307	364	36	0.4	714	1165	2.4	7	42.6		
308	364	36	0.4	714	1165	2.4	28	52		
309	376	24	0.4	714	1165	2.4	3	28		
310	376	24	0.4	714	1165	2.4	7	39		
311	376	24	0.4	714	1165	2.4	28	47		
312	388	12	0.4	714	1165	2.4	3	28		
313	388	12	0.4	714	1165	2.4	7	37.5		
314	388	12	0.4	714	1165	2.4	28	42		
315	400	0	0.4	714	1165	2.4	7	36.1		
316	400	0	0.4	714	1165	2.4	28	40		
317	516	0	0.31	348	1513	2.064	3	54.6	cube 100	[15]
318	426.2	123.75	0.22	795	935	7.974275	3	57.7		
319	426.2	123.75	0.22	795	935	7.974275	7	94.25		
320	426.2	123.75	0.22	795	935	7.974275	28	107.5		
321	467.5	82.5	0.26	765	901	6.875	3	53.3		
322	467.5	82.5	0.26	765	901	6.875	7	87.07		
323	467.5	82.5	0.26	765	901	6.875	28	105.8		
324	508.75	41.25	0.31	739	870	4.95	3	54.9		
325	508.75	41.25	0.31	739	870	4.95	7	75.76		
326	508.75	41.25	0.31	739	870	4.95	28	94.1		
327	508.75	41.25	0.31	739	870	4.95	90	101.2		
328	516	0	0.31	348	1513	2.064	7	75.7		
329	516	0	0.31	348	1513	2.064	28	88.9		
330	516	0	0.31	348	1513	2.064	90	97.7		
331	596	0	0.26	337	1465	3.576	3	60.9		
332	596	0	0.26	337	1465	3.576	7	84.48		
333	596	0	0.26	337	1465	3.576	28	103.2		
334	681	0	0.22	325	1415	6.129	3	62		

335	681	0	0.22	325	1415	6.129	7	86.05		
336	681	0	0.22	325	1415	6.129	28	101.5		
337	500	0	0.38	900	650	3	7	25	cube 150	[16]
338	425	75	0.38	900	650	5	7	32		
339	425	75	0.38	900	650	5	28	62.5		
340	425	75	0.38	900	650	5	56	65		
341	450	50	0.38	900	650	4	7	48		
342	450	50	0.38	900	650	4	28	71		
343	450	50	0.38	900	650	4	56	76		
344	475	25	0.38	900	650	3	7	32.5		
345	475	25	0.38	900	650	3	28	54		
346	475	25	0.38	900	650	3	56	63.5		
347	500	0	0.38	900	650	3	28	43		
348	500	0	0.38	900	650	3	56	49.5		
349	420	0	0.430952	668	1232	0	7	31.75	cube 150	[17]
350	336	84	0.430952	668	1232	0	7	34.25		
351	336	84	0.430952	668	1232	0	28	43.25		
352	336	84	0.430952	668	1232	0	90	46.5		
353	357	63	0.430952	668	1232	0	7	35.2		
354	357	63	0.430952	668	1232	0	28	45.3		
355	357	63	0.430952	668	1232	0	90	48.5		
356	378	42	0.430952	668	1232	0	7	34.25		
357	378	42	0.430952	668	1232	0	28	44.5		
358	378	42	0.430952	668	1232	0	90	48		
359	399	21	0.430952	668	1232	0	7	32.65		
360	399	21	0.430952	668	1232	0	28	42.35		
361	399	21	0.430952	668	1232	0	90	46.5		
362	420	0	0.430952	668	1232	0	28	40.4		
363	420	0	0.430952	668	1232	0	90	44.7		
364	420	0	0.47	948	759	1.638	7	19	cube 100	[18]
365	357	63	0.47	948	759	2.184	7	24.5		
366	357	63	0.37	1007	802	2.604	7	39		
367	357	63	0.47	948	759	2.184	28	40		
368	357	63	0.37	1007	802	2.604	28	57		
369	357	63	0.47	948	759	2.184	90	48		
370	357	63	0.37	1007	802	2.604	90	57.5		
371	357	63	0.47	948	759	2.184	180	51.4		
372	357	63	0.37	1007	802	2.604	180	67		
373	367.5	52.5	0.47	948	759	1.932	7	23		
374	367.5	52.5	0.37	1007	802	2.226	7	41.5		
375	367.5	52.5	0.47	948	759	1.932	28	40		
376	367.5	52.5	0.37	1007	802	2.226	28	58.5		
377	367.5	52.5	0.47	948	759	1.932	90	47.5		

378	367.5	52.5	0.37	1007	802	2.226	90	66.5		
379	367.5	52.5	0.47	948	759	1.932	180	50.05		
380	367.5	52.5	0.37	1007	802	2.226	180	72		
381	378	42	0.47	948	759	1.806	7	26		
382	378	42	0.37	1007	802	1.932	7	38		
383	378	42	0.47	948	759	1.806	28	41		
384	378	42	0.37	1007	802	1.932	28	54		
385	378	42	0.47	948	759	1.806	90	46		
386	378	42	0.37	1007	802	1.932	90	55.05		
387	378	42	0.47	948	759	1.806	180	50		
388	378	42	0.37	1007	802	1.932	180	66		
389	420	0	0.37	1007	802	1.722	7	27		
390	420	0	0.47	948	759	1.638	28	35.5		
391	420	0	0.37	1007	802	1.722	28	32.5		
392	420	0	0.47	948	759	1.638	90	42.5		
393	420	0	0.37	1007	802	1.722	90	60.5		
394	420	0	0.47	948	759	1.638	180	48.5		
395	420	0	0.37	1007	802	1.722	180	64		
396	434.32	0	0.430006	624.77	1264.97	0	7	37	cube 150	[19]
397	304.02	86.86	0.477794	624.77	1264.97	0	7	38		
398	304.02	86.86	0.477794	843.43	822.23	1.52	7	42.4		
399	304.02	86.86	0.477794	624.77	1264.97	0	28	55.2		
400	304.02	86.86	0.477794	843.43	822.23	1.52	28	58		
401	304.02	86.86	0.477794	624.77	1264.97	0	90	69.8		
402	304.02	86.86	0.477794	843.43	822.23	1.52	90	66		
403	325.74	65.12	0.477818	624.77	1264.97	0	7	40.3		
404	325.74	65.12	0.477818	843.43	822.23	1.52	7	44.1		
405	325.74	65.12	0.477818	624.77	1264.97	0	28	57.2		
406	325.74	65.12	0.477818	843.43	822.23	1.52	28	60		
407	325.74	65.12	0.477818	624.77	1264.97	0	90	72.7		
408	325.74	65.12	0.477818	843.43	822.23	1.52	90	67.5		
409	390.89	43.43	0.430006	624.77	1264.97	0	7	41.8		
410	390.89	43.43	0.430006	843.43	822.23	1.52	7	57.9		
411	390.89	43.43	0.430006	624.77	1264.97	0	28	64.5		
412	390.89	43.43	0.430006	843.43	822.23	1.52	28	68.2		
413	390.89	43.43	0.430006	624.77	1264.97	0	90	77.5		
414	390.89	43.43	0.430006	843.43	822.23	1.52	90	79		
415	412.6	21.72	0.430006	624.77	1264.97	0	7	38		
416	412.6	21.72	0.430006	843.43	822.23	1.52	7	52.5		
417	412.6	21.72	0.430006	624.77	1264.97	0	28	54.5		
418	412.6	21.72	0.430006	843.43	822.23	1.52	28	62.4		
419	412.6	21.72	0.430006	624.77	1264.97	0	90	68.8		
420	412.6	21.72	0.430006	843.43	822.23	1.52	90	72.25		

421	434.32	0	0.430006	843.43	822.23	1.52	7	37.6		
422	434.32	0	0.430006	624.77	1264.97	0	28	51.25		
423	434.32	0	0.430006	843.43	822.23	1.52	28	46.2		
424	434.32	0	0.430006	624.77	1264.97	0	90	62.7		
425	434.32	0	0.430006	843.43	822.23	1.52	90	63.75		
426	350	0	0.5	720	1200	0.1995	3	38	cylinder 100*200	[20]
427	280	70	0.5	720	1200	1.4	3	34		
428	280	70	0.5	720	1200	0.595	7	57		
429	280	70	0.5	720	1200	1.4	7	54.4		
430	280	70	0.5	720	1200	0.595	28	75.5		
431	280	70	0.5	720	1200	1.4	28	77.4		
432	280	70	0.5	720	1200	0.595	90	85		
433	280	70	0.5	720	1200	1.4	90	86		
434	315	35	0.5	720	1200	0.49	3	36		
435	315	35	0.5	720	1200	0.6335	3	42.5		
436	315	35	0.5	720	1200	0.49	7	54.4		
437	315	35	0.5	720	1200	0.6335	7	59.4		
438	315	35	0.5	720	1200	0.49	28	74		
439	315	35	0.5	720	1200	0.6335	28	80		
440	315	35	0.5	720	1200	0.49	90	80.4		
441	315	35	0.5	720	1200	0.6335	90	91.4		
442	350	0	0.5	720	1200	0.1995	7	49		
443	350	0	0.5	720	1200	0.1995	28	56		
444	350	0	0.5	720	1200	0.1995	90	67		
445	346	0	0.55	692	1038	0	7	18.8	cylinder 100*200	[21]
446	259.5	86.5	0.55	692	1038	0	7	17		
447	259.5	86.5	0.55	692	1038	0	28	23.9		
448	276.8	69.2	0.55	692	1038	0	7	19		
449	276.8	69.2	0.55	692	1038	0	28	27		
450	294.1	51.9	0.55	692	1038	0	7	22		
451	294.1	51.9	0.55	692	1038	0	28	31.5		
452	311.4	34.6	0.55	692	1038	0	7	20.6		
453	311.4	34.6	0.55	692	1038	0	28	29.5		
454	328.7	17.3	0.55	692	1038	0	7	19		
455	328.7	17.3	0.55	692	1038	0	28	28		
456	346	0	0.55	692	1038	0	28	27.3		
457	510	0	0.317647	806	972	3	7	45.1	cube 150	[22]
458	410	100	0.317647	806	972	4	7	51.4		
459	410	100	0.317647	806	972	4	28	69.6		
460	435	75	0.317647	806	972	3.8	7	54.8		
461	435	75	0.317647	806	972	3.8	28	72.7		
462	460	50	0.317647	806	972	3.6	7	51.9		
463	460	50	0.317647	806	972	3.6	28	70.4		

464	485	25	0.317647	806	972	3.25	7	50.9		
465	485	25	0.317647	806	972	3.25	28	64.6		
466	510	0	0.317647	806	972	3	28	61.9		
467	350	35	0.42	685	1200	0.55825	2	44.6	cube 150	[23]
468	350	70	0.42	650	1200	0.8694	2	45.4		
469	350	35	0.42	685	1200	0.8547	2	46.6		
470	350	70	0.42	650	1200	1.4994	2	49.7		
471	350	35	0.42	685	1200	0.55825	7	73		
472	350	70	0.42	650	1200	0.8694	7	67.1		
473	350	35	0.42	685	1200	0.8547	7	63.3		
474	350	70	0.42	650	1200	1.4994	7	69.7		
475	350	35	0.42	685	1200	0.55825	28	91.6		
476	350	70	0.42	650	1200	0.8694	28	81.5		
477	350	35	0.42	685	1200	0.8547	28	81.4		
478	350	70	0.42	650	1200	1.4994	28	83.7		
479	350	35	0.42	685	1200	0.55825	90	91		
480	350	70	0.42	650	1200	0.8694	90	82.9		
481	350	35	0.42	685	1200	0.8547	90	90.9		
482	350	70	0.42	650	1200	1.4994	90	94.8		
483	450	0	0.26	904	904	5.4	3	48.4	cube 150	[24]
484	384.5	67.5	0.26	904	904	7.232	3	49.9		
485	384.5	67.5	0.26	904	904	7.232	7	72.5		
486	384.5	67.5	0.26	904	904	7.232	28	88.3		
487	405	45	0.26	904	904	6.3	3	48.5		
488	405	45	0.26	904	904	6.3	7	69.3		
489	405	45	0.26	904	904	6.3	28	83.7		
490	427.5	22.5	0.26	904	904	5.4	3	49.8		
491	427.5	22.5	0.26	904	904	5.4	7	65.8		
492	427.5	22.5	0.26	904	904	5.4	28	80.6		
493	450	0	0.26	904	904	5.4	7	59		
494	450	0	0.26	904	904	5.4	28	73.7		
495	550	0	0.25	579.5	177.9	4.23	3	67	cube 150	[25]
496	399.5	70.5	0.35	571.2	175.4	4.31	3	51.1		
497	399.5	70.5	0.35	571.2	175.4	4.31	7	65		
498	399.5	70.5	0.35	571.2	175.4	4.31	28	73		
499	446.5	23.5	0.35	576.5	177	3	3	47.5		
500	446.5	23.5	0.35	576.5	177	3	7	58.4		
501	446.5	23.5	0.35	576.5	177	3	28	66.4		
502	467.5	82.5	0.25	570.3	175.1	8.4	3	64.8		
503	467.5	82.5	0.25	570.3	175.1	8.4	7	79.25		
504	467.5	82.5	0.25	570.3	175.1	8.4	28	83		
505	470	0	0.35	579.1	177.8	1.86	3	45.8		
506	470	0	0.35	579.1	177.8	1.86	7	52		

507	470	0	0.35	579.1	177.8	1.86	28	61.5		
508	522.5	27.5	0.25	576.4	177	5.4	3	67.5		
509	522.5	27.5	0.25	576.4	177	5.4	7	72.5		
510	522.5	27.5	0.25	576.4	177	5.4	28	78.5		
511	550	0	0.25	579.5	177.9	4.23	7	68		
512	550	0	0.25	579.5	177.9	4.23	28	75		
513	380	0	0.5	755	685	7.6	28	33.75	cube 100	[26]
514	266	114	0.5	755	685	7.6	28	36.25		
515	266	114	0.5	755	685	7.6	90	38.55		
516	285	95	0.5	755	685	7.6	28	38.25		
517	285	95	0.5	755	685	7.6	90	41.4		
518	304	76	0.5	755	685	7.6	28	43.7		
519	304	76	0.5	755	685	7.6	90	45.8		
520	323	57	0.5	755	685	7.6	28	44.8		
521	323	57	0.5	755	685	7.6	90	47		
522	342	38	0.5	755	685	7.6	28	39.4		
523	342	38	0.5	755	685	7.6	90	43.1		
524	361	19	0.5	755	685	7.6	28	36.25		
525	361	19	0.5	755	685	7.6	90	38		
526	380	0	0.5	755	685	7.6	90	35		
527	500	0	0.3	725	1087	0.5	3	68.5	cube 100	[27]
528	400	100	0.3	708	1087	1	3	70.8		
529	400	100	0.3	708	1087	1	7	87.6		
530	400	100	0.3	708	1087	1	28	99.6		
531	450	50	0.3	717	1087	0.8	3	85.9		
532	450	50	0.3	717	1087	0.8	7	99.8		
533	475	25	0.3	721	1087	0.6	3	73		
534	475	25	0.3	721	1087	0.6	7	88.2		
535	475	25	0.3	721	1087	0.6	28	103.6		
536	500	0	0.3	725	1087	0.5	7	81.1		
537	500	0	0.3	725	1087	0.5	28	96.5		
538	500	0	0.3	725	1087	0.5	90	102.5		
539	235	0	0.79	700	1200	0.705	1	7.5	cube 100	[28]
540	176.25	58.75	0.79	700	1200	2.767125	1	4		
541	176.25	58.75	0.79	700	1200	2.767125	2	7.5		
542	176.25	58.75	0.79	700	1200	2.767125	7	14		
543	176.25	58.75	0.79	700	1200	2.767125	28	29.5		
544	176.25	58.75	0.79	700	1200	2.767125	60	33		
545	176.25	58.75	0.79	700	1200	2.767125	180	34		
546	188	47	0.79	700	1200	2.1244	1	5		
547	188	47	0.79	700	1200	2.1244	2	9		
548	188	47	0.79	700	1200	2.1244	7	16		
549	188	47	0.79	700	1200	2.1244	28	29.5		

550	188	47	0.79	700	1200	2.1244	60	33		
551	188	47	0.79	700	1200	2.1244	180	34		
552	199.75	35.25	0.79	700	1200	1.578025	1	6		
553	199.75	35.25	0.79	700	1200	1.578025	2	10.5		
554	199.75	35.25	0.79	700	1200	1.578025	7	16.5		
555	199.75	35.25	0.79	700	1200	1.578025	28	29		
556	199.75	35.25	0.79	700	1200	1.578025	60	32.5		
557	199.75	35.25	0.79	700	1200	1.578025	180	34		
558	211.5	23.5	0.79	700	1200	1.33245	1	6.5		
559	211.5	23.5	0.79	700	1200	1.33245	2	11.5		
560	211.5	23.5	0.79	700	1200	1.33245	7	18		
561	211.5	23.5	0.79	700	1200	1.33245	28	28.5		
562	211.5	23.5	0.79	700	1200	1.33245	60	32		
563	211.5	23.5	0.79	700	1200	1.33245	180	34		
564	213.75	71.25	0.65	730	1200	3.29175	1	7.5		
565	213.75	71.25	0.65	730	1200	3.29175	2	13		
566	213.75	71.25	0.65	730	1200	3.29175	7	26.5		
567	213.75	71.25	0.65	730	1200	3.29175	28	44		
568	213.75	71.25	0.65	730	1200	3.29175	60	47.5		
569	213.75	71.25	0.65	730	1200	3.29175	180	49.5		
570	223.25	11.75	0.79	700	1200	0.9823	1	7		
571	223.25	11.75	0.79	700	1200	0.9823	2	11.5		
572	223.25	11.75	0.79	700	1200	0.9823	7	18.5		
573	223.25	11.75	0.79	700	1200	0.9823	28	28		
574	223.25	11.75	0.79	700	1200	0.9823	60	31.5		
575	223.25	11.75	0.79	700	1200	0.9823	180	34.5		
576	228	57	0.65	730	1200	2.622	1	9		
577	228	57	0.65	730	1200	2.622	2	15		
578	228	57	0.65	730	1200	2.622	7	26		
579	228	57	0.65	730	1200	2.622	28	44		
580	228	57	0.65	730	1200	2.622	60	47.5		
581	228	57	0.65	730	1200	2.622	180	49.5		
582	232.5	77.5	0.6	710	1200	3.0225	1	10		
583	232.5	77.5	0.6	710	1200	3.0225	2	16.5		
584	232.5	77.5	0.6	710	1200	3.0225	7	31		
585	232.5	77.5	0.6	710	1200	3.0225	28	50.5		
586	232.5	77.5	0.6	710	1200	3.0225	60	54		
587	232.5	77.5	0.6	710	1200	3.0225	180	55		
588	235	0	0.79	700	1200	0.705	2	12		
589	235	0	0.79	700	1200	0.705	7	18.5		
590	235	0	0.79	700	1200	0.705	28	27.5		
591	235	0	0.79	700	1200	0.705	60	31		
592	235	0	0.79	700	1200	0.705	180	34		

593	242.25	42.75	0.65	730	1200	1.865325	1	9.5		
594	242.25	42.75	0.65	730	1200	1.865325	2	16.5		
595	242.25	42.75	0.65	730	1200	1.865325	7	26.5		
596	242.25	42.75	0.65	730	1200	1.865325	28	43		
597	242.25	42.75	0.65	730	1200	1.865325	60	46.5		
598	242.25	42.75	0.65	730	1200	1.865325	180	49		
599	248	62	0.6	710	1200	2.5792	1	10.5		
600	248	62	0.6	710	1200	2.5792	2	17.5		
601	248	62	0.6	710	1200	2.5792	7	31.5		
602	248	62	0.6	710	1200	2.5792	28	50.5		
603	248	62	0.6	710	1200	2.5792	60	54		
604	248	62	0.6	710	1200	2.5792	180	55		
605	256.5	28.5	0.65	730	1200	1.4877	1	10		
606	256.5	28.5	0.65	730	1200	1.4877	2	17		
607	256.5	28.5	0.65	730	1200	1.4877	7	26.5		
608	256.5	28.5	0.65	730	1200	1.4877	28	41.5		
609	256.5	28.5	0.65	730	1200	1.4877	60	45.5		
610	256.5	28.5	0.65	730	1200	1.4877	180	48		
611	263.5	46.5	0.6	710	1200	1.81815	1	11		
612	263.5	46.5	0.6	710	1200	1.81815	2	19		
613	263.5	46.5	0.6	710	1200	1.81815	7	31.5		
614	263.5	46.5	0.6	710	1200	1.81815	28	49		
615	263.5	46.5	0.6	710	1200	1.81815	60	53		
616	263.5	46.5	0.6	710	1200	1.81815	180	54.5		
617	266.25	88.75	0.52	670	1200	3.860625	1	13		
618	266.25	88.75	0.52	670	1200	3.860625	2	24.5		
619	266.25	88.75	0.52	670	1200	3.860625	7	42.5		
620	266.25	88.75	0.52	670	1200	3.860625	28	62		
621	266.25	88.75	0.52	670	1200	3.860625	60	66		
622	266.25	88.75	0.52	670	1200	3.860625	180	68		
623	270.75	14.25	0.65	730	1200	1.02885	1	10		
624	270.75	14.25	0.65	730	1200	1.02885	2	17		
625	270.75	14.25	0.65	730	1200	1.02885	7	26.5		
626	270.75	14.25	0.65	730	1200	1.02885	28	40		
627	270.75	14.25	0.65	730	1200	1.02885	60	44		
628	270.75	14.25	0.65	730	1200	1.02885	180	47		
629	279	31	0.6	710	1200	1.1997	1	11.5		
630	279	31	0.6	710	1200	1.1997	2	19.5		
631	279	31	0.6	710	1200	1.1997	7	31		
632	279	31	0.6	710	1200	1.1997	28	47		
633	279	31	0.6	710	1200	1.1997	60	51		
634	279	31	0.6	710	1200	1.1997	180	53.5		
635	284	71	0.52	670	1200	3.266	1	14		

636	284	71	0.52	670	1200	3.266	2	25		
637	284	71	0.52	670	1200	3.266	7	42.5		
638	284	71	0.52	670	1200	3.266	28	61.5		
639	284	71	0.52	670	1200	3.266	60	65.5		
640	284	71	0.52	670	1200	3.266	180	67.5		
641	285	0	0.65	730	1200	0.684	1	10.5		
642	285	0	0.65	730	1200	0.684	2	17.5		
643	285	0	0.65	730	1200	0.684	7	26.5		
644	285	0	0.65	730	1200	0.684	28	37		
645	285	0	0.65	730	1200	0.684	60	41		
646	285	0	0.65	730	1200	0.684	180	44.5		
647	294.5	15.5	0.6	710	1200	0.7657	1	12		
648	294.5	15.5	0.6	710	1200	0.7657	2	20		
649	294.5	15.5	0.6	710	1200	0.7657	7	31		
650	294.5	15.5	0.6	710	1200	0.7657	28	44.5		
651	294.5	15.5	0.6	710	1200	0.7657	60	48.5		
652	294.5	15.5	0.6	710	1200	0.7657	180	52		
653	301.75	53.25	0.52	670	1200	2.414	1	15		
654	301.75	53.25	0.52	670	1200	2.414	2	25.5		
655	301.75	53.25	0.52	670	1200	2.414	7	41.5		
656	301.75	53.25	0.52	670	1200	2.414	28	60		
657	301.75	53.25	0.52	670	1200	2.414	60	64.5		
658	301.75	53.25	0.52	670	1200	2.414	180	67		
659	307.5	102.5	0.45	625	1200	4.76625	1	19		
660	307.5	102.5	0.45	625	1200	4.76625	2	31		
661	307.5	102.5	0.45	625	1200	4.76625	7	53.5		
662	307.5	102.5	0.45	625	1200	4.76625	28	73		
663	307.5	102.5	0.45	625	1200	4.76625	60	78		
664	307.5	102.5	0.45	625	1200	4.76625	180	79		
665	310	0	0.6	710	1200	0.403	1	12		
666	310	0	0.6	710	1200	0.403	2	20		
667	310	0	0.6	710	1200	0.403	7	30.5		
668	310	0	0.6	710	1200	0.403	28	41		
669	310	0	0.6	710	1200	0.403	60	45		
670	310	0	0.6	710	1200	0.403	180	49		
671	319.5	35.5	0.52	670	1200	1.5975	1	15.5		
672	319.5	35.5	0.52	670	1200	1.5975	2	26		
673	319.5	35.5	0.52	670	1200	1.5975	7	40.5		
674	319.5	35.5	0.52	670	1200	1.5975	28	57		
675	319.5	35.5	0.52	670	1200	1.5975	60	61		
676	319.5	35.5	0.52	670	1200	1.5975	180	63.5		
677	328	82	0.45	625	1200	3.8704	1	19.5		
678	328	82	0.45	625	1200	3.8704	2	32.5		

679	328	82	0.45	625	1200	3.8704	7	53.5		
680	328	82	0.45	625	1200	3.8704	28	72.5		
681	328	82	0.45	625	1200	3.8704	60	77.5		
682	328	82	0.45	625	1200	3.8704	180	78.5		
683	337.25	17.75	0.52	670	1200	1.315275	1	16		
684	337.25	17.75	0.52	670	1200	1.315275	2	27		
685	337.25	17.75	0.52	670	1200	1.315275	7	40		
686	337.25	17.75	0.52	670	1200	1.315275	28	55		
687	337.25	17.75	0.52	670	1200	1.315275	60	59.5		
688	337.25	17.75	0.52	670	1200	1.315275	180	63		
689	348.5	61.5	0.45	625	1200	2.9274	1	20		
690	348.5	61.5	0.45	625	1200	2.9274	2	33		
691	348.5	61.5	0.45	625	1200	2.9274	7	53.5		
692	348.5	61.5	0.45	625	1200	2.9274	28	71		
693	348.5	61.5	0.45	625	1200	2.9274	60	75.5		
694	348.5	61.5	0.45	625	1200	2.9274	180	78		
695	355	0	0.52	670	1200	0.3905	1	16		
696	355	0	0.52	670	1200	0.3905	2	26		
697	355	0	0.52	670	1200	0.3905	7	38.5		
698	355	0	0.52	670	1200	0.3905	28	50		
699	355	0	0.52	670	1200	0.3905	60	54		
700	355	0	0.52	670	1200	0.3905	180	58		
701	369	41	0.45	625	1200	2.4723	1	20.5		
702	369	41	0.45	625	1200	2.4723	2	33.5		
703	369	41	0.45	625	1200	2.4723	7	50		
704	369	41	0.45	625	1200	2.4723	28	66.5		
705	369	41	0.45	625	1200	2.4723	60	71.5		
706	369	41	0.45	625	1200	2.4723	180	73.5		
707	389.5	20.5	0.45	625	1200	1.6359	1	20.5		
708	389.5	20.5	0.45	625	1200	1.6359	2	33		
709	389.5	20.5	0.45	625	1200	1.6359	7	49		
710	389.5	20.5	0.45	625	1200	1.6359	28	64		
711	389.5	20.5	0.45	625	1200	1.6359	60	68.5		
712	389.5	20.5	0.45	625	1200	1.6359	180	72		
713	410	0	0.45	625	1200	0.861	1	20.5		
714	410	0	0.45	625	1200	0.861	2	32		
715	410	0	0.45	625	1200	0.861	7	47.5		
716	410	0	0.45	625	1200	0.861	28	59		
717	410	0	0.45	625	1200	0.861	60	63.5		
718	410	0	0.45	625	1200	0.861	180	68		
719	330	0	0.6	710	1240	0	7	18.9	cube 150	[29]
720	297	33	0.6	710	1240	0	7	17		
721	280.5	49.5	0.6	710	1240	0	7	14.9		

722	264	66	0.6	710	1240	0	7	12.4		
723	440	0	0.45	600	1240	0	7	30.5		
724	396	44	0.45	600	1240	0	7	30.2		
725	374	66	0.45	600	1240	0	7	32.7		
726	352	88	0.45	600	1240	0	7	24.7		
727	440	0	0.45	600	1240	0	7	22.4		
728	396	44	0.45	600	1240	0	7	24.5		
729	374	66	0.45	600	1240	0	7	20.6		
730	330	0	0.6	710	1240	0	14	22.2		
731	297	33	0.6	710	1240	0	14	22.2		
732	280.5	49.5	0.6	710	1240	0	14	21		
733	264	66	0.6	710	1240	0	14	18.8		
734	440	0	0.45	600	1240	0	14	33.7		
735	396	44	0.45	600	1240	0	14	35.9		
736	374	66	0.45	600	1240	0	14	40.4		
737	352	88	0.45	600	1240	0	14	32.1		
738	440	0	0.45	600	1240	0	14	27.1		
739	396	44	0.45	600	1240	0	14	32.6		
740	374	66	0.45	600	1240	0	14	30.5		
741	330	0	0.6	710	1240	0	28	23.9		
742	297	33	0.6	710	1240	0	28	26.6		
743	280.5	49.5	0.6	710	1240	0	28	25		
744	264	66	0.6	710	1240	0	28	23.4		
745	440	0	0.45	600	1240	0	28	40.6		
746	396	44	0.45	600	1240	0	28	40.9		
747	374	66	0.45	600	1240	0	28	48		
748	352	88	0.45	600	1240	0	28	39.7		
749	440	0	0.45	600	1240	0	28	29.7		
750	396	44	0.45	600	1240	0	28	37.9		
751	374	66	0.45	600	1240	0	28	36.4		
752	330	0	0.6	710	1240	0	90	28.1		
753	297	33	0.6	710	1240	0	90	31.1		
754	280.5	49.5	0.6	710	1240	0	90	31.5		
755	264	66	0.6	710	1240	0	90	29.6		
756	440	0	0.45	600	1240	0	90	44		
757	396	44	0.45	600	1240	0	90	44.8		
758	374	66	0.45	600	1240	0	90	53.7		
759	352	88	0.45	600	1240	0	90	46.8		
760	440	0	0.45	600	1240	0	90	38.5		
761	396	44	0.45	600	1240	0	90	45.6		
762	374	66	0.45	600	1240	0	90	43.5		
763	330	0	0.6	710	1240	0	180	29.1		
764	297	33	0.6	710	1240	0	180	31.8		
765	280.5	49.5	0.6	710	1240	0	180	33.1		

766	264	66	0.6	710	1240	0	180	33.6		
767	440	0	0.45	600	1240	0	180	44.8		
768	396	44	0.45	600	1240	0	180	45.4		
769	374	66	0.45	600	1240	0	180	54.6		
770	352	88	0.45	600	1240	0	180	47.6		
771	440	0	0.45	600	1240	0	180	43.3		
772	396	44	0.45	600	1240	0	180	48		
773	374	66	0.45	600	1240	0	180	50.3		
774	450	0	0.4	670	1100	1.125	7	67.42	cube 100	[30]
775	427.5	22.5	0.4	670	1100	2.25	7	73.25		
776	405	45	0.4	670	1100	3.375	7	78.43		
777	382.5	67.5	0.4	670	1100	4.5	7	86.7		
778	360	90	0.4	670	1100	4.5	7	84.78		
779	450	0	0.4	670	1100	1.125	28	84.89		
780	427.5	22.5	0.4	670	1100	2.25	28	89.05		
781	405	45	0.4	670	1100	3.375	28	98.86		
782	382.5	67.5	0.4	670	1100	4.5	28	99.74		
783	360	90	0.4	670	1100	4.5	28	94.5		
784	450	0	0.4	670	1100	1.125	56	91.16		
785	427.5	22.5	0.4	670	1100	2.25	56	96.23		
786	405	45	0.4	670	1100	3.375	56	100.6		
787	382.5	67.5	0.4	670	1100	4.5	56	100.54		
788	360	90	0.4	670	1100	4.5	56	96.84		
789	450	0	0.4	670	1100	1.125	90	92.28		
790	427.5	22.5	0.4	670	1100	2.25	90	97.12		
791	405	45	0.4	670	1100	3.375	90	101.15		
792	382.5	67.5	0.4	670	1100	4.5	90	103.67		
793	360	90	0.4	670	1100	4.5	90	100.87		
794	372	0	0.5	698	1238	0	28	31.9	cube 150	[31]
795	352.4	18.6	0.5	698	1238	0	28	35.84		
796	334.8	37.2	0.5	698	1238	0	28	38.81		
797	316.2	55.8	0.5	698	1238	0	28	32.58		
798	297.6	74.4	0.5	698	1238	0	28	32		
799	400	0	0.55	880	800	8	28	40.7	cube 100	[32]
800	380	20	0.55	880	800	8	28	48.3		
801	360	40	0.55	880	800	8	28	51.8		
802	340	60	0.55	880	800	8	28	54.5		
803	320	80	0.55	880	800	8	28	57.2		
804	300	100	0.55	880	800	8	28	53.7		
805	280	120	0.55	880	800	8	28	51.5		
806	589	0	0.28	925	808	6.61	28	98.6	cylinder 100*200	[33]
807	547	0	0.35	913	798	5.86	28	88.1		
808	492	55	0.35	915	800	7.1	28	98.2		
809	500	0	0.38	818	908	4	7	61	cube 100	[34]

810	475	25	0.38	818	908	4	7	56		
811	450	50	0.38	818	908	4.6	7	57		
812	425	75	0.38	818	908	4.8	7	53		
813	500	0	0.38	818	908	4	28	68		
814	475	25	0.38	818	908	4	28	63		
815	450	50	0.38	818	908	4.6	28	66		
816	425	75	0.38	818	908	4.8	28	70		
817	500	0	0.38	818	908	4	180	79		
818	475	25	0.38	818	908	4	180	71		
819	450	50	0.38	818	908	4.6	180	72		
820	425	75	0.38	818	908	4.8	180	74		
821	600	0	0.38	750	600	12	7	21.22	cube 150	[35]
822	570	30	0.38	750	600	12	7	25.23		
823	540	60	0.38	750	600	12	7	27.56		
824	510	90	0.38	750	600	12	7	32.77		
825	480	120	0.38	750	600	12	7	30.45		
826	450	150	0.38	750	600	12	7	29.76		
827	600	0	0.38	750	600	12	14	25.6		
828	570	30	0.38	750	600	12	14	29.11		
829	540	60	0.38	750	600	12	14	36.81		
830	510	90	0.38	750	600	12	14	39.16		
831	480	120	0.38	750	600	12	14	37.3		
832	450	150	0.38	750	600	12	14	32.33		
833	600	0	0.38	750	600	12	21	32.81		
834	570	30	0.38	750	600	12	21	35.06		
835	540	60	0.38	750	600	12	21	42.09		
836	510	90	0.38	750	600	12	21	45.87		
837	480	120	0.38	750	600	12	21	44.6		
838	450	150	0.38	750	600	12	21	41.11		
839	600	0	0.38	750	600	12	28	39.15		
840	570	30	0.38	750	600	12	28	40.55		
841	540	60	0.38	750	600	12	28	46.79		
842	510	90	0.38	750	600	12	28	49.08		
843	480	120	0.38	750	600	12	28	47.9		
844	450	150	0.38	750	600	12	28	45.75		
845	575	0	0.3	554	1190	24	7	88	cube 100	[36]
846	563.5	11.5	0.3	554	1190	24	7	94.9		
847	552	23	0.3	554	1190	24	7	94.8		
848	540.5	34.5	0.3	554	1190	24	7	93.7		
849	529	46	0.3	554	1190	24	7	97.2		
850	517.5	57.5	0.3	554	1190	24	7	101.3		
851	506	69	0.3	554	1190	24	7	95.5		
852	494.5	80.5	0.3	554	1190	24	7	97.1		
853	483	92	0.3	554	1190	24	7	97.4		

854	460	115	0.3	554	1190	24	7	94.1		
855	431.25	143.75	0.3	554	1190	24	7	95.7		
856	575	0	0.3	554	1190	24	28	104		
857	336.42	83.58	0.37	964.94	868.45	10.43	28	74.8	cylinder 100*200	[37]
858	455.52	24.48	0.37	914.42	822.98	4	28	74		
859	393.75	56.25	0.4	920.98	828.88	4.57	28	76.5		
860	393.75	56.25	0.4	920.98	828.88	4.57	28	77.8		
861	384.48	95.52	0.43	867.89	781.1	4.86	28	72.5		
862	393.75	56.25	0.45	890.19	801.18	3.29	28	67.3		
863	393.75	56.25	0.35	951.77	856.59	15.43	28	78.3		
864	350	50	0.4	970	873.63	8.14	28	62.1		
865	398.58	21.42	0.37	971.17	874.05	8.71	28	54.1		
866	337.5	112.5	0.4	915.35	823.82	7.33	28	79.9		
867	336.42	83.58	0.43	930.46	837.41	6.14	28	68.9		
868	393.75	56.25	0.4	920.98	828.88	5.29	28	79.1		
869	455.52	24.48	0.43	875	787.5	2.57	28	61.1		
870	398.58	21.42	0.43	936.68	843.02	3.57	28	58.1		
871	393.57	56.25	0.4	920.98	828.88	4.71	28	77.8		
872	393.75	56.25	0.4	920.98	828.88	4.57	28	80.2		
873	384.48	95.52	0.37	907.3	816.57	9.57	28	82.3		
874	437.5	62.5	0.4	871.27	784.14	4.29	28	82.2		
875	393.75	56.25	0.4	920.98	828.88	5.29	28	81.9		
876	450	0	0.4	926.62	833.95	2.33	28	57		
877	480	0	0.32	979	817	3.51	3	20	cube 100	[38]
878	456	24	0.32	987	820	3.6	3	22		
879	432	48	0.32	987	815	3.72	3	23.9		
880	408	72	0.32	980	815	3.34	3	24.1		
881	384	96	0.32	989	805	2.8	3	21.3		
882	480	0	0.32	979	817	3.51	7	33		
883	456	24	0.32	987	820	3.6	7	35.6		
884	432	48	0.32	987	815	3.72	7	37.2		
885	408	72	0.32	980	815	3.34	7	35.1		
886	384	96	0.32	989	805	2.8	7	33.6		
887	480	0	0.32	979	817	3.51	14	35		
888	456	24	0.32	987	820	3.6	14	36.9		
889	432	48	0.32	987	815	3.72	14	39.3		
890	408	72	0.32	980	815	3.34	14	39.7		
891	384	96	0.32	989	805	2.8	14	36		
892	480	0	0.32	979	817	3.51	28	45		
893	456	24	0.32	987	820	3.6	28	46.1		
894	432	48	0.32	987	815	3.72	28	49.8		
895	408	72	0.32	980	815	3.34	28	50.3		
896	384	96	0.32	989	805	2.8	28	46		
897	480	0	0.32	979	817	3.51	56	53		

898	456	24	0.32	987	820	3.6	56	52		
899	432	48	0.32	987	815	3.72	56	55		
900	408	72	0.32	980	815	3.34	56	56.3		
901	384	96	0.32	989	805	2.8	56	54.6		
902	550	0	0.4	1502	0	6.82	1	25.8	cube 100	[39]
903	523	27.5	0.4	1498	0	8.2	1	21		
904	495	55	0.4	1494	0	10	1	32.7		
905	468	82.5	0.4	1488	0	11.8	1	33		
906	550	0	0.4	1502	0	6.82	3	57.9		
907	523	27.5	0.4	1498	0	8.2	3	58.6		
908	495	55	0.4	1494	0	10	3	52.9		
909	468	82.5	0.4	1488	0	11.8	3	55.9		
910	550	0	0.4	1502	0	6.82	7	66.5		
911	523	27.5	0.4	1498	0	8.2	7	71.5		
912	495	55	0.4	1494	0	10	7	73.7		
913	468	82.5	0.4	1488	0	11.8	7	75.7		
914	550	0	0.4	1502	0	6.82	14	71.6		
915	523	27.5	0.4	1498	0	8.2	14	77.7		
916	495	55	0.4	1494	0	10	14	81.8		
917	468	82.5	0.4	1488	0	11.8	14	81.9		
918	550	0	0.4	1502	0	6.82	28	72.7		
919	523	27.5	0.4	1498	0	8.2	28	80.7		
920	495	55	0.4	1494	0	10	28	85.1		
921	468	82.5	0.4	1488	0	11.8	28	89.7		
922	380	0	0.45	656	1169	0.76	7	23.7	cube 100	[40]
923	361	19	0.45	656	1169	0.76	7	27.6		
924	342	38	0.45	656	1169	0.76	7	31.4		
925	323	57	0.45	656	1169	0.76	7	34.8		
926	304	76	0.45	656	1169	0.76	7	33.1		
927	285	95	0.45	656	1169	0.76	7	32.1		
928	266	114	0.45	656	1169	0.76	7	30.4		
929	380	0	0.45	656	1169	0.76	28	39.4		
930	361	19	0.45	656	1169	0.76	28	42.7		
931	342	38	0.45	656	1169	0.76	28	46.1		
932	323	57	0.45	656	1169	0.76	28	49.4		
933	304	76	0.45	656	1169	0.76	28	48.4		
934	285	95	0.45	656	1169	0.76	28	47.3		
935	266	114	0.45	656	1169	0.76	28	45.1		
936	500	0	0.38	650	900	5	7	45	cube 100	[41]
937	450	50	0.38	650	900	7.5	7	56.3		
938	500	0	0.38	650	900	5	28	52.5		
939	450	50	0.38	650	900	7.5	28	61.5		
940	440	0	0.34	1172	580	10.17	28	70	cube 150	[42]
941	418	22	0.34	1172	580	10.17	28	59		

942	396	44	0.34	1172	580	10.17	28	62		
943	374	66	0.34	1172	580	10.17	28	62.7		
944	352	88	0.34	1172	580	10.17	28	55		
945	425	0	0.4	1172	580	3.7	28	63		
946	403.75	21.25	0.4	1172	580	3.7	28	57		
947	382.5	42.5	0.4	1172	580	3.7	28	44		
948	361.25	63.75	0.4	1172	580	3.7	28	47.5		
949	340	85	0.4	1172	580	3.7	28	38.5		
950	400	0	0.45	1100	720	0	28	53.5		
951	380	20	0.45	1100	720	1.28	28	46.5		
952	360	40	0.45	1100	720	1.28	28	40.05		
953	340	60	0.45	1100	720	1.28	28	31		
954	320	80	0.45	1100	720	1.28	28	36		
955	465	0	0.4	840	820	6.98	7	48.4	cube 100	[43]
956	441.75	13.4	0.4	840	820	6.63	7	50.6		
957	465	0	0.4	840	820	6.98	28	58.4		
958	441.75	13.4	0.4	840	820	6.63	28	62.2		
959	400	0	0.55	880	800	8	7	36	cube 100	[44]
960	380	20	0.55	880	800	8	7	38.75		
961	360	40	0.55	880	800	8	7	42		
962	340	60	0.55	880	800	8	7	43.9		
963	320	80	0.55	880	800	8	7	44.4		
964	300	100	0.55	880	800	8	7	41.8		
965	280	120	0.55	880	800	8	7	40		
966	400	0	0.55	880	800	8	28	41		
967	380	20	0.55	880	800	8	28	47		
968	360	40	0.55	880	800	8	28	52.3		
969	340	60	0.55	880	800	8	28	54.5		
970	320	80	0.55	880	800	8	28	57.2		
971	300	100	0.55	880	800	8	28	53.7		
972	280	120	0.55	880	800	8	28	51.7		
973	400	0	0.55	880	800	8	90	43.75		
974	380	20	0.55	880	800	8	90	51		
975	360	40	0.55	880	800	8	90	52.9		
976	340	60	0.55	880	800	8	90	56.1		
977	320	80	0.55	880	800	8	90	57.2		
978	300	100	0.55	880	800	8	90	55.03		
979	280	120	0.55	880	800	8	90	52.7		
980	570	0	0.28	830.8	863.1	5.13	28	68.6	cube 150	[45]
981	541.5	28.5	0.28	823.9	857.8	6.84	28	83.9		
982	484.5	85.5	0.28	812.3	849.2	8.55	28	86.8		

Table S2: Experimental database of f_{st} of concrete with metakaolin

S.No.	Cement (kg/m^3)	Metakaolin (kg/m^3)	w/b	Fine aggregate (kg/m^3)	Coarse aggregate (kg/m^3)	SP (kg/m^3)	Days	f_{st} (MPa)	Cylinder (mm)	Ref.
1	533.33	0	0.300002	677	1201	2.13	28	4.76	100*200	[3]
2	506.67	26.67	0.299996	666	1182	2.93	28	4.78		
3	480	53.33	0.300002	655	1163	3.47	28	5.19		
4	453.33	80	0.300002	645	1144	4.26	28	4.69		
5	490	0	0.32	967	817	2.47	28	3.04	150*300	[5]
6	466	24.5	0.32	967	815	4.57	28	3.244		
7	441	49	0.32	975	805	7.02	28	3.38		
8	417	73.5	0.32	980	800	7.35	28	3.36		
9	392	98	0.32	985	795	9.96	28	3.12		
10	460	0	0.38	940	815	1.73	28	2.8		
11	437	23	0.38	947	810	3.31	28	2.94		
12	414	46	0.38	958	800	3.6	28	3.02		
13	391	69	0.38	964	790	4.89	28	3.04		
14	368	92	0.38	970	781	4.89	28	2.8		
15	435	0	0.45	920	815	1.63	28	2.56		
16	414	21.8	0.45	925	805	1.95	28	2.72		
17	392	43.5	0.45	933	800	2.93	28	2.82		
18	370	65.2	0.45	940	795	3.26	28	2.8		
19	348	87	0.45	945	785	3.91	28	2.59		
20	450	0	0.35	957	852	7.9	1	1.75	100*200	[7]
21	405	45	0.35	952	848	10.1	1	2.2		
22	360	90	0.35	948	844	12.4	1	2.35		
23	350	0	0.55	960	854	3.5	1	1.2		
24	315	35	0.55	956	851	4.4	1	1.2		
25	280	70	0.55	953	848	6.1	1	1.25		
26	450	0	0.35	957	852	7.9	3	2.27		
27	405	45	0.35	952	848	10.1	3	2.4		
28	360	90	0.35	948	844	12.4	3	2.5		
29	350	0	0.55	960	854	3.5	3	1.6		
30	315	35	0.55	956	851	4.4	3	1.7		
31	280	70	0.55	953	848	6.1	3	2		
32	450	0	0.35	957	852	7.9	7	2.35		
33	405	45	0.35	952	848	10.1	7	2.5		
34	360	90	0.35	948	844	12.4	7	2.6		
35	350	0	0.55	960	854	3.5	7	2.15		
36	315	35	0.55	956	851	4.4	7	2.25		
37	280	70	0.55	953	848	6.1	7	2.3		
38	450	0	0.35	957	852	7.9	28	2.9		
39	405	45	0.35	952	848	10.1	28	3		
40	360	90	0.35	948	844	12.4	28	3.1		

41	350	0	0.55	960	854	3.5	28	2.35		
42	315	35	0.55	956	851	4.4	28	2.5		
43	280	70	0.55	953	848	6.1	28	2.55		
44	450	0	0.35	957	852	7.9	90	3.04		
45	405	45	0.35	952	848	10.1	90	3.4		
46	360	90	0.35	948	844	12.4	90	3.6		
47	350	0	0.55	960	854	3.5	90	2.4		
48	315	35	0.55	956	851	4.4	90	2.65		
49	280	70	0.55	953	848	6.1	90	2.9		
50	450	0	0.35	957	852	7.9	120	3.3		
51	405	45	0.35	952	848	10.1	120	3.7		
52	360	90	0.35	948	844	12.4	120	3.85		
53	350	0	0.55	960	854	3.5	120	2.7		
54	315	35	0.55	956	851	4.4	120	3		
55	280	70	0.55	953	848	6.1	120	3.25		
56	437.78	0	0.449998	643.68	1104.36	0	28	2.79	150*300	[8]
57	415.89	21.89	0.449998	643.68	1104.36	0	28	2.81		
58	394	43.78	0.449998	643.68	1104.36	0	28	2.9		
59	372.11	65.67	0.449998	643.68	1104.36	0	28	3.04		
60	350.22	87.56	0.449998	643.68	1104.36	0	28	2.95		
61	512	0	0.319141	594	1037	5.12	28	5.02	150*300	[11]
62	512	51.2	0.290128	594	1037	5.12	28	5.26		
63	512	76.8	0.277514	594	1037	5.12	28	5.48		
64	512	102.4	0.265951	594	1037	5.12	28	5.15		
65	512	153.6	0.245493	594	1037	5.12	28	5.04		
66	512	204.8	0.227958	594	1037	5.12	28	4.43		
67	512	256	0.21276	594	1037	5.12	28	4.14		
68	400	0	0.5	545	1012	0	7	2.7	150*300	[12]
69	400	10	0.554268	490.5	1012	0	7	2.85		
70	400	20	0.605952	436	1012	0	7	2.92		
71	400	30	0.655233	317.8	1012	0	7	3		
72	400	40	0.702273	327	1012	0	7	3.15		
73	400	50	0.747222	272.5	1012	0	7	2.7		
74	400	0	0.5	545	1012	0	28	3		
75	400	10	0.554268	490.5	1012	0	28	3.1		
76	400	20	0.605952	436	1012	0	28	3.25		
77	400	30	0.655233	317.8	1012	0	28	3.4		
78	400	40	0.702273	327	1012	0	28	3.55		
79	400	50	0.747222	272.5	1012	0	28	2.96		
80	400	0	0.5	545	1012	0	91	3.45		
81	400	10	0.554268	490.5	1012	0	91	3.5		
82	400	20	0.605952	436	1012	0	91	3.6		
83	400	30	0.655233	317.8	1012	0	91	3.7		
84	400	40	0.702273	327	1012	0	91	3.9		

85	400	50	0.747222	272.5	1012	0	91	3.25		
86	500	0	0.38	900	650	3	28	3.5	150*300	[16]
87	475	25	0.38	900	650	3	28	3.65		
88	450	50	0.38	900	650	4	28	5.72		
89	425	75	0.38	900	650	5	28	5.3		
90	420	0	0.430952	668	1232	0	28	3.2	150*300	[17]
91	399	21	0.430952	668	1232	0	28	3.27		
92	378	42	0.430952	668	1232	0	28	3.44		
93	357	63	0.430952	668	1232	0	28	3.4		
94	336	84	0.430952	668	1232	0	28	3.25		
95	434.32	0	0.430006	624.77	1264.97	0	7	3.1	100*200	[19]
96	412.6	21.72	0.430006	624.77	1264.97	0	7	3.5		
97	390.89	43.43	0.430006	624.77	1264.97	0	7	3.8		
98	325.74	65.12	0.477818	624.77	1264.97	0	7	3.55		
99	304.02	86.86	0.477794	624.77	1264.97	0	7	3.2		
100	434.32	0	0.430006	843.43	822.23	1.52	7	3.17		
101	412.6	21.72	0.430006	843.43	822.23	1.52	7	3.67		
102	390.89	43.43	0.430006	843.43	822.23	1.52	7	3.75		
103	325.74	65.12	0.477818	843.43	822.23	1.52	7	3.83		
104	304.02	86.86	0.477794	843.43	822.23	1.52	7	3.89		
105	434.32	0	0.430006	624.77	1264.97	0	28	3.95		
106	412.6	21.72	0.430006	624.77	1264.97	0	28	4.15		
107	390.89	43.43	0.430006	624.77	1264.97	0	28	4.56		
108	325.74	65.12	0.477818	624.77	1264.97	0	28	4.2		
109	304.02	86.86	0.477794	624.77	1264.97	0	28	4		
110	434.32	0	0.430006	843.43	822.23	1.52	28	4.12		
111	412.6	21.72	0.430006	843.43	822.23	1.52	28	4.63		
112	390.89	43.43	0.430006	843.43	822.23	1.52	28	4.75		
113	325.74	65.12	0.477818	843.43	822.23	1.52	28	4.8		
114	304.02	86.86	0.477794	843.43	822.23	1.52	28	4.83		
115	434.32	0	0.430006	624.77	1264.97	0	90	4.55		
116	412.6	21.72	0.430006	624.77	1264.97	0	90	4.67		
117	390.89	43.43	0.430006	624.77	1264.97	0	90	4.9		
118	325.74	65.12	0.477818	624.77	1264.97	0	90	4.76		
119	304.02	86.86	0.477794	624.77	1264.97	0	90	4.61		
120	434.32	0	0.430006	843.43	822.23	1.52	90	4.72		
121	412.6	21.72	0.430006	843.43	822.23	1.52	90	5.12		
122	390.89	43.43	0.430006	843.43	822.23	1.52	90	5.28		
123	325.74	65.12	0.477818	843.43	822.23	1.52	90	5.32		
124	304.02	86.86	0.477794	843.43	822.23	1.52	90	5.38		
125	550	0	0.25	579.5	177.9	4.23	3	4.6	150*300	[25]
126	522.5	27.5	0.25	576.4	177	5.4	3	4.7		
127	467.5	82.5	0.25	570.3	175.1	8.4	3	3.7		
128	470	0	0.35	579.1	177.8	1.86	3	2.25		

129	446.5	23.5	0.35	576.5	177	3	3	2.75		
130	399.5	70.5	0.35	571.2	175.4	4.31	3	3.55		
131	550	0	0.25	579.5	177.9	4.23	7	4.72		
132	522.5	27.5	0.25	576.4	177	5.4	7	5.3		
133	467.5	82.5	0.25	570.3	175.1	8.4	7	5.5		
134	470	0	0.35	579.1	177.8	1.86	7	3.2		
135	446.5	23.5	0.35	576.5	177	3	7	3.2		
136	399.5	70.5	0.35	571.2	175.4	4.31	7	4.07		
137	550	0	0.25	579.5	177.9	4.23	28	5.27		
138	522.5	27.5	0.25	576.4	177	5.4	28	5.8		
139	467.5	82.5	0.25	570.3	175.1	8.4	28	5.88		
140	470	0	0.35	579.1	177.8	1.86	28	3.5		
141	446.5	23.5	0.35	576.5	177	3	28	3.92		
142	399.5	70.5	0.35	571.2	175.4	4.31	28	4.4		
143	380	0	0.5	755	685	7.6	28	2.75	150*300	[26]
144	361	19	0.5	755	685	7.6	28	2.75		
145	342	38	0.5	755	685	7.6	28	3		
146	323	57	0.5	755	685	7.6	28	3.2		
147	304	76	0.5	755	685	7.6	28	3.3		
148	285	95	0.5	755	685	7.6	28	3.12		
149	266	114	0.5	755	685	7.6	28	2.8		
150	450	0	0.4	670	1100	1.125	7	3.59	150*150	[30]
151	427.5	22.5	0.4	670	1100	2.25	7	4.15		
152	405	45	0.4	670	1100	3.375	7	4.98		
153	382.5	67.5	0.4	670	1100	4.5	7	5.58		
154	360	90	0.4	670	1100	4.5	7	4.4		
155	450	0	0.4	670	1100	1.125	28	4.19		
156	427.5	22.5	0.4	670	1100	2.25	28	4.89		
157	405	45	0.4	670	1100	3.375	28	5.2		
158	382.5	67.5	0.4	670	1100	4.5	28	5.42		
159	450	0	0.4	670	1100	1.125	56	4.39		
160	427.5	22.5	0.4	670	1100	2.25	56	5.73		
161	405	45	0.4	670	1100	3.375	56	5.78		
162	382.5	67.5	0.4	670	1100	4.5	56	5.4		
163	360	90	0.4	670	1100	4.5	56	5.64		
164	450	0	0.4	670	1100	1.125	90	5.15		
165	427.5	22.5	0.4	670	1100	2.25	90	5.66		
166	405	45	0.4	670	1100	3.375	90	5.82		
167	382.5	67.5	0.4	670	1100	4.5	90	5.62		
168	480	0	0.32	979	817	3.51	28	3.3	150*300	[38]
169	456	24	0.32	987	820	3.6	28	3.3		
170	432	48	0.32	987	815	3.72	28	3.5		
171	408	72	0.32	980	815	3.34	28	3.6		
172	384	96	0.32	989	805	2.8	28	3.2		

173	500	0	0.38	650	900	5	7	2.9	100*200	[41]
174	450	50	0.38	650	900	7.5	7	3.36		
175	500	0	0.38	650	900	5	28	3.2		
176	450	50	0.38	650	900	7.5	28	3.74		
177	465	0	0.4	840	820	6.98	7	2.49	100*200	[43]
178	441.75	13.4	0.4	840	820	6.63	7	2.79		
179	465	0	0.4	840	820	6.98	28	3.13		
180	441.75	13.4	0.4	840	820	6.63	28	3.36		
181	400	0	0.55	880	800	8	7	2.2	150*300	[44]
182	380	20	0.55	880	800	8	7	2.33		
183	360	40	0.55	880	800	8	7	2.64		
184	340	60	0.55	880	800	8	7	2.76		
185	320	80	0.55	880	800	8	7	2.88		
186	300	100	0.55	880	800	8	7	2.7		
187	280	120	0.55	880	800	8	7	2.71		
188	400	0	0.55	880	800	8	28	2.38		
189	380	20	0.55	880	800	8	28	2.74		
190	360	40	0.55	880	800	8	28	2.92		
191	340	60	0.55	880	800	8	28	3.15		
192	320	80	0.55	880	800	8	28	3.31		
193	300	100	0.55	880	800	8	28	3.1		
194	280	120	0.55	880	800	8	28	2.95		
195	400	0	0.55	880	800	8	90	2.57		
196	380	20	0.55	880	800	8	90	2.8		
197	360	40	0.55	880	800	8	90	3.02		
198	340	60	0.55	880	800	8	90	3.24		
199	320	80	0.55	880	800	8	90	3.6		
200	300	100	0.55	880	800	8	90	3.15		
201	280	120	0.55	880	800	8	90	3.08		
202	570	0	0.28	830.8	863.1	5.13	28	4.04	150*300	[45]
203	541.5	28.5	0.28	823.9	857.8	6.84	28	4.82		
204	484.5	85.5	0.28	812.3	849.2	8.55	28	5.32		

Table S3: Experimental database of FS of concrete with metakaolin

S.No.	Cement (kg/m^3)	Metakaolin (kg/m^3)	w/b	Fine aggregate (kg/m^3)	Coarse aggregate (kg/m^3)	SP (kg/m^3)	Days	FS (MPa)	Ref.
1	434.32	0	0.430006	624.77	1264.97	0	7	4.5	[19]
2	412.6	21.72	0.430006	624.77	1264.97	0	7	5.45	
3	390.89	43.43	0.430006	624.77	1264.97	0	7	6.2	
4	325.74	65.12	0.477818	624.77	1264.97	0	7	5.8	
5	304.02	86.86	0.477794	624.77	1264.97	0	7	5.6	

6	434.32	0	0.430006	843.43	822.23	1.52	7	4.6	
7	412.6	21.72	0.430006	843.43	822.23	1.52	7	5.625	
8	390.89	43.43	0.430006	843.43	822.23	1.52	7	6.4	
9	325.74	65.12	0.477818	843.43	822.23	1.52	7	6.62	
10	304.02	86.86	0.477794	843.43	822.23	1.52	7	6.7	
11	434.32	0	0.430006	624.77	1264.97	0	28	5.4	
12	412.6	21.72	0.430006	624.77	1264.97	0	28	6.8	
13	390.89	43.43	0.430006	624.77	1264.97	0	28	7.6	
14	325.74	65.12	0.477818	624.77	1264.97	0	28	7.4	
15	304.02	86.86	0.477794	624.77	1264.97	0	28	7.22	
16	434.32	0	0.430006	843.43	822.23	1.52	28	5.5	
17	412.6	21.72	0.430006	843.43	822.23	1.52	28	6.9	
18	390.89	43.43	0.430006	843.43	822.23	1.52	28	7.8	
19	325.74	65.12	0.477818	843.43	822.23	1.52	28	8.4	
20	304.02	86.86	0.477794	843.43	822.23	1.52	28	8.8	
21	434.32	0	0.430006	624.77	1264.97	0	90	6.75	
22	412.6	21.72	0.430006	624.77	1264.97	0	90	8.8	
23	390.89	43.43	0.430006	624.77	1264.97	0	90	10.4	
24	325.74	65.12	0.477818	624.77	1264.97	0	90	9.8	
25	304.02	86.86	0.477794	624.77	1264.97	0	90	9.6	
26	434.32	0	0.430006	843.43	822.23	1.52	90	6.8	
27	412.6	21.72	0.430006	843.43	822.23	1.52	90	9	
28	390.89	43.43	0.430006	843.43	822.23	1.52	90	10.5	
29	325.74	65.12	0.477818	843.43	822.23	1.52	90	10.6	
30	304.02	86.86	0.477794	843.43	822.23	1.52	90	10.75	
31	450	0	0.4	670	1100	1.125	7	5.88	[30]
32	427.5	22.5	0.4	670	1100	2.25	7	7.83	
33	405	45	0.4	670	1100	3.375	7	7.21	
34	382.5	67.5	0.4	670	1100	4.5	7	6.62	
35	360	90	0.4	670	1100	4.5	7	7.63	
36	450	0	0.4	670	1100	1.125	28	7.68	
37	427.5	22.5	0.4	670	1100	2.25	28	7.96	
38	405	45	0.4	670	1100	3.375	28	7.91	
39	382.5	67.5	0.4	670	1100	4.5	28	7.16	
40	360	90	0.4	670	1100	4.5	28	7.7	
41	450	0	0.4	670	1100	1.125	56	8.15	
42	427.5	22.5	0.4	670	1100	2.25	56	8.18	
43	405	45	0.4	670	1100	3.375	56	8.45	
44	382.5	67.5	0.4	670	1100	4.5	56	8.9	
45	360	90	0.4	670	1100	4.5	56	8.17	
46	450	0	0.4	670	1100	1.125	90	8.2	
47	427.5	22.5	0.4	670	1100	2.25	90	8.19	
48	405	45	0.4	670	1100	3.375	90	8.6	
49	382.5	67.5	0.4	670	1100	4.5	90	8.85	

50	360	90	0.4	670	1100	4.5	90	7.43	
51	437.78	0	0.449998	643.68	1104.36	0	28	5.84	[8]
52	415.89	21.89	0.449998	643.68	1104.36	0	28	6.12	
53	394	43.78	0.449998	643.68	1104.36	0	28	6.32	
54	372.11	65.67	0.449998	643.68	1104.36	0	28	6.73	
55	350.22	87.56	0.449998	643.68	1104.36	0	28	6.45	
56	510	0	0.317647	806	972	3	28	6.3	[22]
57	485	25	0.317647	806	972	3.25	28	6.6	
58	460	50	0.317647	806	972	3.6	28	7	
59	435	75	0.317647	806	972	3.8	28	7.2	
60	410	100	0.317647	806	972	4	28	6.8	
61	570	0	0.28	830.8	863.1	5.13	28	6.4	[45]
62	541.5	28.5	0.28	823.9	857.8	6.84	28	6.8	
63	484.5	85.5	0.28	812.3	849.2	8.55	28	7.2	

References

1. Akin, O.O.; Ocholi, A.; Abejide, O.S.; Obari, J.A. Prediction of the Compressive Strength of Concrete Admixed with Metakaolin Using Gene Expression Programming. *Adv. Civ. Eng.* **2020**, *2020*, 8883412.
2. Qian, X.; Li, Z. The relationships between stress and strain for high-performance concrete with metakaolin. *Cem. Concr. Res.* **2001**, *31*, 1607–1611.
3. Dinakar, P.; Sahoo, P.K.; Sriram, G. Effect of metakaolin content on the properties of high strength concrete. *Int. J. Concr. Struct. Mater.* **2013**, *7*, 215–223.
4. Poon, C.-S.; Kou, S.; Lam, L. Compressive strength, chloride diffusivity and pore structure of high performance metakaolin and silica fume concrete. *Constr. Build. Mater.* **2006**, *20*, 858–865.
5. Madandoust, R.; Mousavi, S.Y. Fresh and hardened properties of self-compacting concrete containing metakaolin. *Constr. Build. Mater.* **2012**, *35*, 752–760.
6. Ramezanianpour, A.; Jovein, H.B. Influence of metakaolin as supplementary cementing material on strength and durability of concretes. *Constr. Build. Mater.* **2012**, *30*, 470–479.
7. Güneyisi, E.; Gesoğlu, M.; Mermerdaş, K. Improving strength, drying shrinkage, and pore structure of concrete using metakaolin. *Mater. Struct.* **2008**, *41*, 937–949.
8. John, N. Strength properties of metakaolin admixed concrete. *Int. J. Sci. Res. Publ.* **2013**, *3*, 1–7.
9. Khatib, J. Metakaolin concrete at a low water to binder ratio. *Constr. Build. Mater.* **2008**, *22*, 1691–1700.
10. Gill, A.S.; Siddique, R. Strength and micro-structural properties of self-compacting concrete containing metakaolin and rice husk ash. *Constr. Build. Mater.* **2017**, *157*, 51–64.
11. El-Din, H.K.S.; Eisa, A.S.; Aziz, B.H.A.; Ibrahim, A. Mechanical performance of high strength concrete made from high volume of Metakaolin and hybrid fibers. *Constr. Build. Mater.* **2017**, *140*, 203–209.
12. Rashad, A.M. A preliminary study on the effect of fine aggregate replacement with metakaolin on strength and abrasion resistance of concrete. *Constr. Build. Mater.* **2013**, *44*, 487–495.
13. Siddique, R.; Kadri, E.-H. Effect of metakaolin and foundry sand on the near surface characteristics of concrete. *Constr. Build. Mater.* **2011**, *25*, 3257–3266.
14. Wang, G.; Kong, Y.; Shui, Z.; Li, Q.; Han, J. Experimental investigation on chloride diffusion and binding in concrete containing metakaolin. *Corros. Eng. Sci. Technol.* **2014**, *49*, 282–286.
15. Dinakar, P.; Manu, S. Concrete mix design for high strength self-compacting concrete using metakaolin. *Mater. Des.* **2014**, *60*, 661–668.
16. Kavitha, O.; Shanthi, V.; Arulraj, G.P.; Sivakumar, P. Fresh, micro-and macrolevel studies of metakaolin blended self-compacting concrete. *Appl. Clay Sci.* **2015**, *114*, 370–374.
17. Muduli, R.; Mukharjee, B.B. Effect of incorporation of metakaolin and recycled coarse aggregate on properties of concrete. *J. Clean. Prod.* **2019**, *209*, 398–414.

18. Joshaghani, A.; Moeini, M.A.; Balapour, M. Evaluation of incorporating metakaolin to evaluate durability and mechanical properties of concrete. *Adv. Concr. Constr.* **2017**, *5*, 241.
19. Lenka, S.; Panda, K. Effect of metakaolin on the properties of conventional and self compacting concrete. *Adv. Concr. Constr.* **2017**, *5*, 031.
20. Badogiannis, E.; Tsivilis, S.; Papadakis, V.; Chaniotakis, E. The effect of metakaolin on concrete properties. In Proceedings of the International Congress on Challenges of Concrete Construction in Innovation and Development in Concrete Materials and Construction, Scotland, UK, 9–11 September 2002; pp. 81–89.
21. Saand, A.; Keerio, M.A.; Bangwar, D.K. Effect of soorh metakaolin on concrete compressive strength and durability. *Eng. Technol. Appl. Sci. Res.* **2017**, *7*, 2210–2214.
22. Narmatha, M.; Felixkala, T. Analyse the mechanical properties of metakaolin using as a partial replacement of cement in concrete. *Int. J. Adv. Res. Ideas Innov. Technol.* **2017**, *3*, 25–30.
23. Badogiannis, E.; Tsivilis, S. Exploitation of poor Greek kaolins: Durability of metakaolin concrete. *Cem. Concr. Compos.* **2009**, *31*, 128–133.
24. Bonakdar, A.; Bakhshi, M.; Ghalibafian, M. Properties of High-performance Concrete ContainingHigh Reactivity Metakaolin. In Proceedings of 7th International Symposium on Utilization of High-Strength/High-Performance Concrete, Washington, DC, USA,1 Junaury 2005 pp. 228–295.
25. Güneyisi, E.; Gesoğlu, M.; Karaoğlu, S.; Mermerdaş, K. Strength, permeability and shrinkage cracking of silica fume and metakaolin concretes. *Constr. Build. Mater.* **2012**, *34*, 120–130.
26. Kannan, V. Strength and durability performance of self compacting concrete containing self-combusted rice husk ash and metakaolin. *Constr. Build. Mater.* **2018**, *160*, 169–179.
27. Poon, C.; Kou, S.; Lam, L. Pore size distribution of high performance metakaolin concrete. *J. Wuhan Univ. Technol. -Mater. Sci. Ed.* **2002**, *17*, 42–46.
28. Meddah, M.S.; Ismail, M.A.; El-Gamal, S.; Fitriani, H. Performances evaluation of binary concrete designed with silica fume and metakaolin. *Constr. Build. Mater.* **2018**, *166*, 400–412.
29. Ferreira, R.; Castro-Gomes, J.; Costa, P.; Malheiro, R. Effect of metakaolin on the chloride ingress properties of concrete. *KSCE J. Civ. Eng.* **2016**, *20*, 1375–1384.
30. Shafiq, N.; Nuruddin, M.F.; Khan, S.U.; Ayub, T. Calcined kaolin as cement replacing material and its use in high strength concrete. *Constr. Build. Mater.* **2015**, *81*, 313–323.
31. Dubey, S.; Chandak, R.; Yadav, R. Experimental study of concrete with metakaolin as partial replacement of OPC. *Int J Adv. Eng Res Sci* **2015**, *2*, 38–40.
32. Kannan, V.; Ganesan, K. Chloride and chemical resistance of self compacting concrete containing rice husk ash and metakaolin. *Constr. Build. Mater.* **2014**, *51*, 225–234.
33. Akcay, B.; Tasdemir, M.A. Performance evaluation of silica fume and metakaolin with identical finenesses in self compacting and fiber reinforced concretes. *Constr. Build. Mater.* **2018**, *185*, 436–444.
34. Bumanis, G.; Bajare, D.; Korjakins, A. Durability of high strength self compacting concrete with metakaolin containing waste. In *Key Engineering Materials*; Trans Tech Publications Ltd: Zurich, Switzerland, 2016; pp. 65–70.
35. Ofuyatan, O.M.; Olowofoyeku, A.M.; Edeki, S.; Oluwafemi, J.; Ajao, A.; David, O. Incorporation of silica fume and metakaolin on self compacting concrete. *J. Phys. Conf. Ser.* **2019**, *1378*, 042089.
36. Ženíšek, M.; Vlach, T.; Laiblová, L. Dosage of Metakaolin in high performance concrete. In *Key Engineering Materials*; Trans Tech Publications Ltd: Zurich, Switzerland, 2017; pp. 311–315.
37. Abouhussien, A.A.; Hassan, A.A. Application of statistical analysis for mixture design of high-strength self-consolidating concrete containing metakaolin. *J. Mater. Civ. Eng.* **2014**, *26*, 04014016.
38. Sharbatdar, M.K.; Abbasi, M.; Fakharian, P. Improving the properties of self-compacted concrete with using combined silica fume and metakaolin. *Period. Polytech. Civ. Eng.* **2020**, *64*, 535–544.
39. Güneyisi, E.; Gesoğlu, M. Properties of self-compacting mortars with binary and ternary cementitious blends of fly ash and metakaolin. *Mater. Struct.* **2008**, *41*, 1519–1531.
40. Kesavraman, S. Studies on Metakaolin based banana fibre reinforced concrete. *Int. J. Civ. Eng. Technol.* **2017**, *8*, 532–543.
41. Al-Oran, A.A.A.; Safiee, N.A.; Nasir, N.A.M. Fresh and hardened properties of self-compacting concrete using metakaolin and GGBS as cement replacement. *Eur. J. Environ. Civ. Eng.* **2019**, *26*, 379–392.
42. Salimi, J.; Ramezanianpour, A.M.; Moradi, M.J. Studying the effect of low reactivity metakaolin on free and restrained shrinkage of high performance concrete. *J. Build. Eng.* **2020**, *28*, 101053.
43. Zoe, Y.; Hanif, I.; Adzmier, H.; Eyzati, H.; Syuhaili, M.N. Strength of Self-Compacting Concrete Containing Metakaolin and Nylon Fiber. *IOP Conf. Ser. : Earth Environ. Sci.* **2020**, *498*, 012047.
44. Kannan, V.; Ganesan, K. Evaluation of mechanical and permeability related properties of self compacting concrete containing metakaolin. *Sci. Res. Essays* **2012**, *7*, 4081–4091.

45. Güneyisi, E.; Gesoğlu, M.; Qays, M.A.; Mermerdaş, K.; İpek, S. Fracture properties of high strength metakaolin and silica fume concretes. In Proceedings of the 3rd International Conference on Chemical, Civil and Environmental Engineering (CCEE-2016), Antalya, Turkey, 20–21 April 2016.
